# Effectiveness of discharge planning interventions on health-related outcomes among postpartum women: a systematic review and meta-analysis

**DOI:** 10.3389/fpubh.2026.1733799

**Published:** 2026-03-23

**Authors:** Qiaomin Huang, Ao Peng, Zheng’ai Cui, Ziyin Huang, Dongmei Duan, Xinyu Cao, Ying’ai Cui, Lihua Huang

**Affiliations:** 1 School of Nursing, Guangdong Medical University, Dongguan, China; 2 School of Humanities and Management, Guangdong Medical University, Dongguan, China; 3 Dongguan Maternal and Child Health Care Hospital, Dongguan, China

**Keywords:** discharge planning, health outcomes, meta-analysis, postpartum women, systematic review

## Abstract

**Background:**

Postpartum women frequently experience psychological distress, physical morbidities, and sub-optimal maternal–neonatal care skills. Structured discharge planning may mitigate these problems, yet its overall effectiveness and the active ingredients underpinning benefit remain unclear.

**Objective:**

To synthesis randomized and quasi-experimental evidence on the effectiveness of discharge planning interventions for improving postpartum women’s health outcomes and to identify intervention characteristics that modify this effectiveness.

**Methods:**

Nine electronic databases (including PubMed, Web of Science, PsycINFO, CINAHL, EMBASE, Cochrane Library, CNKI, Wanfang, and SinoMed) were searched from inception to 10 May 2025. Randomized controlled trials (RCTs) and quasi-experimental studies evaluating discharge planning interventions aimed at postpartum women were eligible. Two reviewers independently screened records, assessed risk of bias using the Cochrane RoB 2.0 and ROBINS-I tools, and extracted data. The odds ratio (OR) with its 95% confidence interval (CI) was used as the primary statistical measure. For continuous variables, the standardized mean difference (SMD) was used. Statistical heterogeneity was quantified using the *I*^2^ statistic.

**Results:**

Twenty-one studies (13 RCTs and 8 quasi-experimental studies) involving 33,096 participants from nine countries were included. Discharge planning produced moderate-to-large improvements in: mental health – depressive symptoms (SMD = −0.64; 95% CI: −1.27, −0.01) and anxiety (SMD = −1.29; 95% CI: −2.22, −0.37); self-care and neonatal-care competence (OR = 2.34; 95% CI: 1.20, 4.58); and breastfeeding self-efficacy (SMD = 2.86; 95% CI: 1.63, 4.08). Interventions integrating predischarge education, post-discharge telephone follow-up, and information-based communication platforms tended to demonstrate larger effect sizes.

**Conclusion:**

This systematic review and meta-analysis indicates that structured discharge planning programs implemented in hospital-based maternity wards during the early postpartum period can improve maternal mental health, self-care capacity, and maternal–neonatal outcomes. The evidence, derived from both randomized controlled trials and pragmatic quasi-experimental studies conducted in routine clinical settings, suggests that ward- or institution-level discharge interventions are effective and scalable strategies for optimizing postnatal transitions.

**Systematic review registration:**

https://www.crd.york.ac.uk/PROSPERO/view/CRD420251055810, registered 20/05/2025.

## 1 Introduction

Postpartum health constitutes a critical determinant of maternal, neonatal and societal trajectories, integrating physical recuperation, psychological stability, functional restoration and social reintegration (1). While postpartum mental disorders—particularly major depressive episode (7%) and any depressive symptoms (10–20%) (2)—receive predominant attention, a substantial proportion of women develop persistent, medium- to long-term (>6 weeks postpartum) morbidities directly attributable to parturition and delivery. Epidemiologic syntheses identify the following as the most common sequelae, expressed as pooled prevalence estimates: dyspareunia (35%), lumbopelvic pain (32%), urinary incontinence (8–31%), anxiety disorders (9–24%), anal incontinence (19%), depressive disorders (11–17%), tokophobia (6–15%), perineal pain (11%), and secondary infertility (11%) (3). Moreover, many mothers lack the knowledge, skills and confidence for self-care, newborn care and effective breastfeeding (4, 5). Compounding this, the hospital-to-home transition is routinely under-supported: shortened stays, fragmented follow-up and scarce mental-health services leave mothers without timely guidance, jeopardizing both neonatal outcomes and maternal recovery (6, 7). These converging deficits underscore the urgent need for continuity-focused interventions.

Faced with this multidimensional and inequitable postpartum morbidity, health systems require structured interventions that extend care beyond the hospital ward. Discharge planning is a key mechanism for ensuring postnatal care continuity. Through integrated predischarge assessment, tailored education and scheduled follow-up, it mitigates the care discontinuity women experience on transition from hospital to home, thereby improving short- and long-term outcomes (8, 9). Despite this recognized strategy, 72% of mothers report unmet needs within 72 h of discharge, most frequently inadequate neonatal-care skills, uncertainty about self-recovery and insufficient psychological support (10, 11).

Nevertheless, the evidence base for discharge-planning efficacy remains piecemeal and pivotal questions unresolved. The principal limitation is the narrow constellation of end-points: extant studies almost exclusively rely on readily quantifiable somatic metrics—readmission rates, complication incidence, or length-of-stay (12, 13)—thereby failing to encapsulate women’s holistic health trajectories or functional recovery. Consequently, psychological-functional domains—postpartum depression, anxiety, parenting stress, self-efficacy, self-care capacity, neonatal-care proficiency, and breastfeeding confidence and sustainability—remain scantly appraised (14–16). Such a myopic lens eclipses the comprehensive dividends of discharge planning. A rigorously effective intervention must elicit concomitant, synergistic improvements across physiological, psychological, and functional dimensions to substantiate its full value.

Although a recent scoping review by Nguyen et al. (4) mapped the typology and implementation features of maternal self-care interventions across the perinatal continuum, no systematic review has yet focused specifically on postpartum discharge planning. The available evidence remains fragmented; moreover, the effectiveness, cost-effectiveness, and scalability of discharge-planning interventions—particularly in low- and middle-income countries that account for 70% of global births—have not been synthesized (17).

In the decade 2009–2020, hemorrhage (27%), indirect obstetric conditions (23%), and hypertensive disorders (16%) remained the leading causes of the estimated 287,000 annual maternal deaths worldwide, with the majority of complications occurring during the critical postpartum period (18). While these preventable deaths and morbidities are disproportionately concentrated in low- and middle-income countries (LMICs), they highlight a universal challenge: the vulnerability of the transition from hospital to home. This global health disparity provides a strong impetus to evaluate structured interventions for bridging the care gap. Against this backdrop, a rigorous systematic review is required to determine whether comprehensive postpartum discharge planning—by improving continuity of care, early recognition of complications, and timely referral—can serve as a low-cost, high-impact strategy to narrow persistent inequalities in postnatal survival and maternal health.

## 2 Materials and methods

This study was conducted in accordance with the Cochrane Handbook for Systematic Reviews of Interventions for systematic review and meta-analysis (19). The Preferred Reporting Items for Systematic Reviews and Meta-Analyses (PRISMA) (20) guidelines were strictly adhered to. Additionally, the study protocol was registered in PROSPERO (ID: CRD420251055810).

### 2.1 Literature search strategy

The search period spanned from the inception of each database to May 10, 2025. Six English-language databases (PubMed, Web of Science, PsycINFO, CINAHL, EMBASE, and CENTRAL) and three Chinese databases (CNKI, Wanfang, and SinoMed) were systematically searched. Appropriate subject headings and search terms were combined and adjusted according to the indexing systems of the respective databases. The detailed search strategy for each database is provided in Supplementary File S1.

EndNote 20 was utilized for managing, categorizing, and removing duplicate records. From May 11 to June 1, 2025, two researchers (Q.H. and O.P.) independently screened the titles and abstracts of the retrieved literature to identify relevant studies. Following this initial screening, the full texts of the selected articles were thoroughly reviewed to determine their eligibility for inclusion. Any discrepancies that arose during this process were resolved through discussion with a third researcher (Y.C.), ensuring consensus was reached.

### 2.2 Inclusion criteria

Studies included in this review met the following criteria: (a) Postpartum women during the puerperium period; (b) Structured discharge planning, discharge education, or discharge guidance programs provided during hospitalization or the early postpartum period (≤6 weeks); (c) Studies reporting any measurable maternal or neonatal health-related outcomes, including but not limited to physical health indicators (e.g., postpartum recovery, postpartum complications), mental health outcomes (e.g., depressive or anxiety symptoms), maternal and infant outcomes (e.g., neonatal health status, breastfeeding outcomes), healthcare utilization, quality of life, symptom management, and self-care ability. (d) RCTs or quasi-experimental designs.

### 2.3 Exclusion criteria

Studies were excluded if they met any of the following criteria: (a) Those in which discharge planning, discharge education, or discharge guidance was part of a multifaceted intervention, making it impossible to isolate their individual effects; (b) Articles lacking sufficient statistical data to calculate effect sizes or those without detailed intervention descriptions; (c) Reports not published in English or Chinese.

While RCTs were prioritized to ensure high internal validity, quasi-experimental studies were also included to capture large-scale, real-world evidence from diverse healthcare settings, thereby enhancing the external validity and geographic reach of the synthesis.

### 2.4 Study selection

Two researchers (Q.H. and O.P.) independently screened the titles, abstracts, and full texts of retrieved records against the eligibility criteria. Discrepancies were resolved through discussion or consultation with a third researcher apps (Y.C.). In addition to the PICOS criteria, during the full-text screening phase, studies judged to have critically flawed methodological features that would preclude meaningful interpretation of intervention effects were excluded. These critical flaws included, but were not limited to, the absence of a control group, extremely high (>50%) and substantially imbalanced attrition rates between groups, or study designs fundamentally inappropriate for causal inference. This preliminary methodological screening was applied solely to exclude studies lacking a minimum methodological framework and was conducted prior to the formal risk of bias assessment using standardized tools.

### 2.5 Data extraction

From June 2 to July 1, 2025, the six authors (Q.H., O.P., C.Z., Z.H., D.D., and X.C.) used a standardized data extraction form to collect information from the selected studies. The form included the following parameters: author, publication year, country, study design, participants, intervention, evaluation time points, and main measures.

### 2.6 Quality assessment

Two researchers (Q.H. and Y.C.) rigorously evaluated the quality of all included studies. In cases of disagreement, a third researcher (L.H.) was consulted to make the final determination regarding risk of bias ratings. The Cochrane Risk of Bias Tool (RoB 2) (21) was employed for RCTs, while the Risk Of Bias In Non-randomized Studies of Interventions (ROBINS-I) tool (22) was utilized for quasi-experimental studies. Subsequently, the research team graded the overall quality (low, moderate, or high risk) of the included studies based on the risk of bias assessment and examined the potential impact of bias on the interpretation of study results.

### 2.7 Data synthesis and analysis

Data analysis was conducted using Stata 18. For binary categorical variables (such as self-care and neonatal care competence), the odds ratio (OR) is used as the effect size pooling indicator. For continuous variables (such as depression, anxiety, and breastfeeding self-efficacy), since the measurement tools used in each study are not uniform, the standardized mean difference (SMD) is adopted for pooling. Maternal depressive symptoms were assessed using either the Self-Rating Depression Scale (SDS) or the Edinburgh Postnatal Depression Scale (EPDS). Maternal anxiety was assessed using either the Self-Rating Anxiety Scale (SAS) or the State–Trait Anxiety Inventory for Adults (STAI-AD). Breastfeeding self-efficacy was assessed using the Chinese version of the Breastfeeding Self-Efficacy Scale (BSES), the Breastfeeding Self-Efficacy Scale (BSS), or the Breastfeeding Self-Efficacy Scale-Short Form (BSES-SF). Although these scales differ in item structure and scoring ranges, they measure similar underlying constructs and have demonstrated acceptable reliability and validity in postpartum populations. For meta-analysis, either a random-effects model (*I*^2^ ≥ 50%) or a fixed-effects model (*I*^2^ < 50%) was selected based on the degree of heterogeneity (assessed via the *I*^2^ statistic). If heterogeneity exceeded 50%, subgroup analyses were conducted to explore potential sources of variation.

## 3 Results

### 3.1 Studies selection

**Figure 1:**
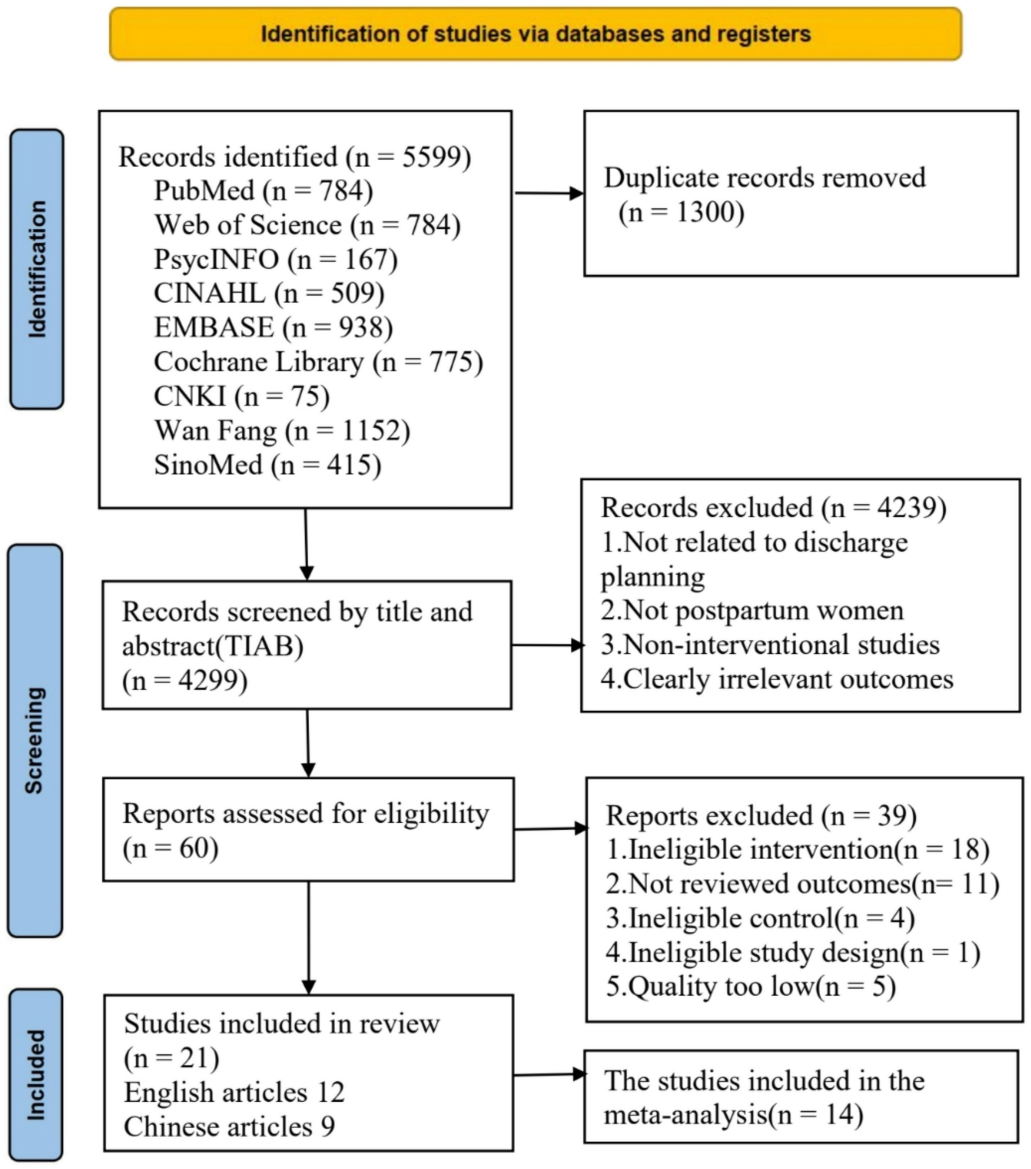
PRISMA flow diagram.

The preliminary database search yielded 5,599 records, of which 1,300 duplicates were removed. A total of 4,299 records were screened by title and abstract. During this stage, 4,239 records were excluded because they were not related to discharge planning interventions, did not involve postpartum women, were non-interventional studies, or reported clearly irrelevant outcomes. Sixty full-text articles were subsequently assessed for eligibility. Of these, 39 articles were excluded for the following reasons: ineligible intervention (*n* = 18), Not reviewed outcomes (*n* = 11), Ineligible control (*n* = 4), Ineligible study design (*n* = 1), Quality too low (*n* = 5). Ultimately, 21 studies were included in the systematic review and meta-analysis, comprising 12 English-language studies and 9 Chinese-language studies. The study selection process is illustrated in Figure 1.

Among the 21 studies included in the systematic review, 14 studies were eligible for quantitative synthesis. 7 studies were not included in the meta-analysis for methodological reasons. First, 6 studies reporting breastfeeding outcomes were excluded from pooling due to substantial variability in follow-up time points (23–28), which ranged from prior to discharge and at discharge to 48 h post-discharge, 1–3 months, 6 weeks, and 4 months postpartum. Pooling outcomes across such widely dispersed assessment times would introduce considerable clinical heterogeneity and compromise the interpretability of the pooled estimates. Furthermore, one study reporting neonatal complications and readmission outcomes was not included in the meta-analysis because fewer than three studies provided comparable data for these endpoints (*K* < 3) (29), precluding a statistically robust quantitative synthesis.

### 3.2 Study characteristics

The 21 studies in this review comprised controlled trials published between 2007 and 2025, encompassing research from multiple countries: 11 from China (23–25, 28, 30–36), 2 each from the United States (37, 38), Turkey (39, 40), and India (29, 41), and 1 each from Brazil (42), Spain (27), Italy (26), and the United Kingdom (43). Among the included studies, 13 were RCTs (24, 25, 28–37, 39), and 8 were quasi-experimental studies (23, 26, 27, 38, 40–43). 13 studies enrolled both multiparous and primiparous women; 7 studies included only primiparous women, and 1 study exclusively involved preterm parturients. A total of 33,096 participants were included, with 18,335 (55.4%) in the intervention group and 14,761 (44.6%) in the control group. The main characteristics included in the study are described in Supplementary File S2.

Regarding outcome measures, 11 studies assessed the psychological health of postpartum women (32–40, 42, 43), focusing on depression, anxiety, parenting confidence, role competence, and breastfeeding self-efficacy. These studies evaluated the impact of discharge planning interventions on postpartum women’s mental health using psychological scales, questionnaires, and other assessment tools. Additionally, 15 studies evaluated physiological indicators in postpartum women, emphasizing maternal complications, neonatal complications, length of hospital stay, and exclusive breastfeeding rates (23–26, 28, 29, 31, 32, 34, 36, 37, 40–43). The assessment of these psychological and physiological indicators contributes to understanding the overall health impact of discharge planning on postpartum women.

Regarding intervention strategies, the included studies employed various implementation approaches, which were primarily categorized into predischarge education (E), post-discharge telephone follow-up (T), and information exchange platforms (P). Specifically, predischarge education provided a foundational knowledge base, while telephone follow-up offered personalized clinical guidance. Information exchange platforms served to enhance this support further, providing a continuous, round-the-clock channel for consultation and peer interaction. Most interventions were combinations of these components rather than single strategies, reflecting a multimodal approach to postpartum care.

### 3.3 Risk of bias assessment

In the RCTs, 4 studies were rated as having a low risk of bias, 8 studies raised some concerns, and 1 study was classified as high risk. Specifically, 5 studies raised some concerns regarding the randomization process—1 study due to the absence of baseline comparisons (33), and 4 studies due to insufficient information on sequence generation and allocation concealment (24, 25, 31, 34). Four studies were rated as having some concerns due to the lack of participant blinding and deviations from the intended interventions likely caused by the study context (29, 30, 34, 35). One study raised some concerns regarding bias due to missing outcome data (37), as it reported dropouts but explicitly explained the reasons, suggesting that the missing data might not have influenced the outcomes. One study was rated as high risk for bias due to missing outcome data (29), given its high dropout rate and lack of evidence confirming that missing data did not affect the results; thus, the missingness was likely dependent on the true values.

**Figure 2:**
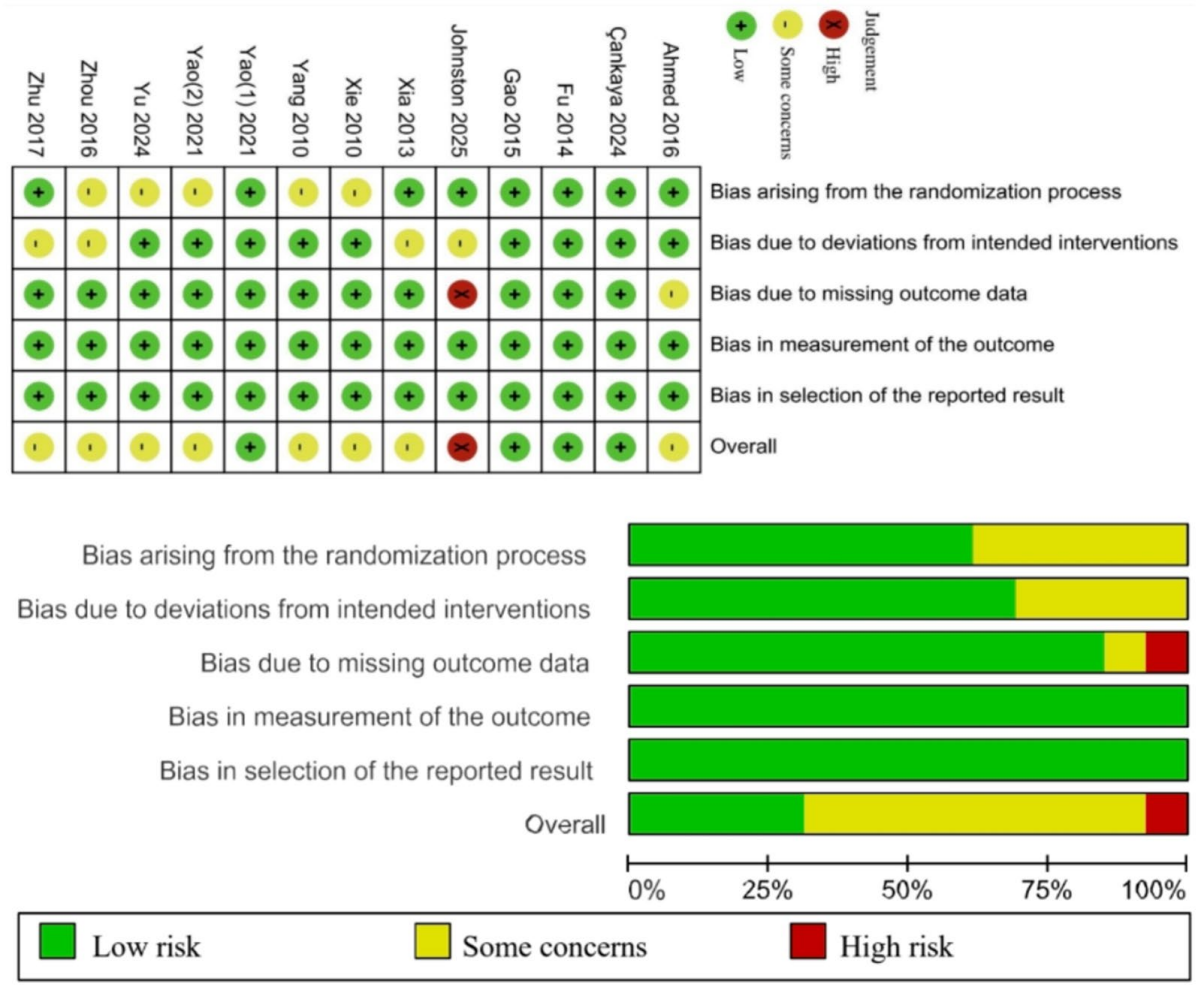
Summary of risk of bias of randomized controlled trials.

**Figure 3:**
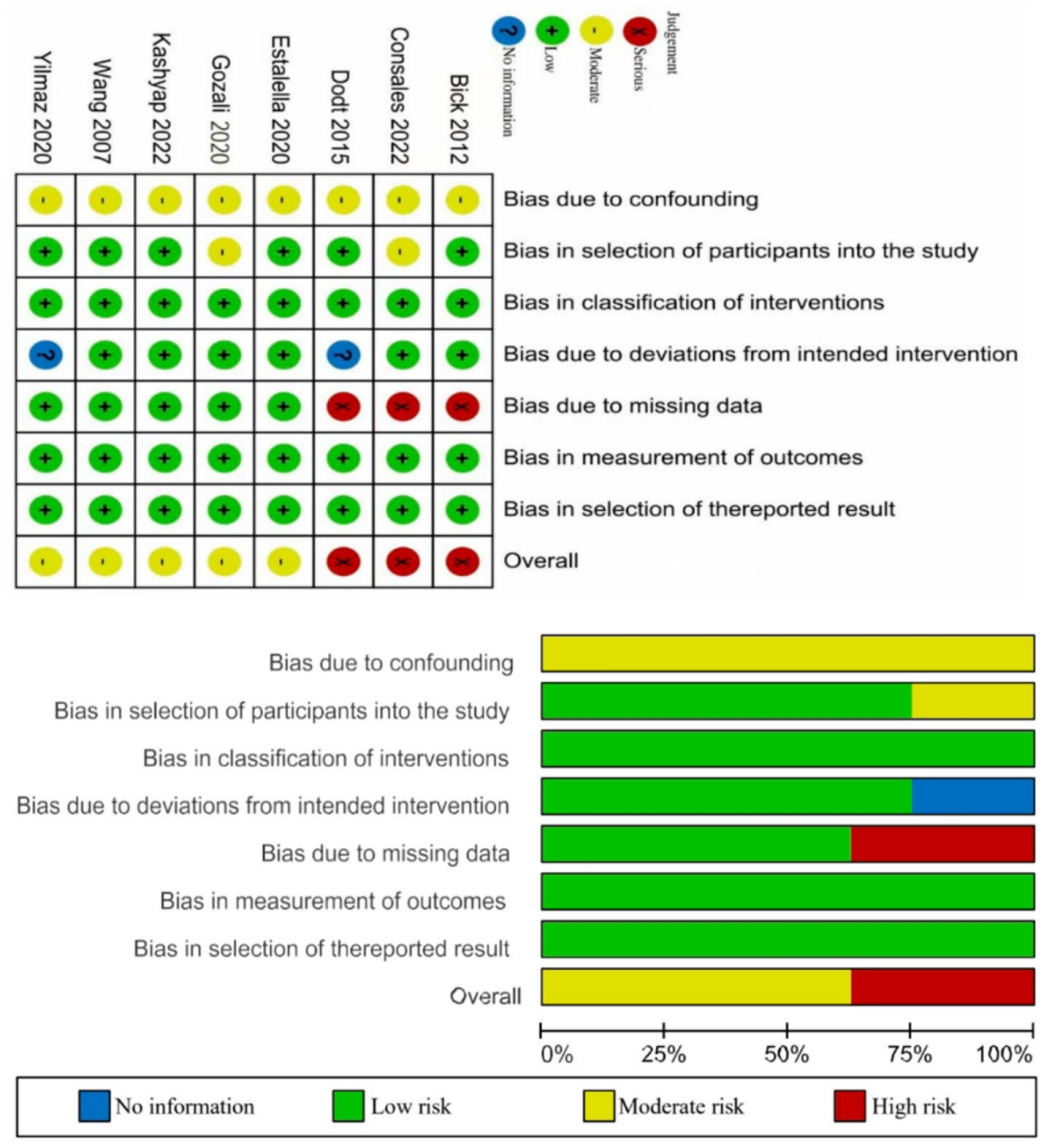
Summary of risk of bias of quasi-experimental studies.

Among the 8 quasi-experimental studies, 3 studies were assessed as having a serious overall risk of bias, while 5 studies had a moderate risk. Due to the nature of their designs, all 8 studies were considered to have a moderate risk of bias arising from non-randomization. 2 studies had a moderate risk of bias in participant selection due to language-based or voluntary participation criteria (26, 38). 2 studies provided insufficient information to assess deviations from intended interventions (40, 42). Notably, 3 studies exhibited a risk of bias due to missing data, given their high participant attrition rates (26, 42, 43). However, all 8 studies demonstrated a low risk of bias in intervention classification, outcome measurement, and selective reporting. The risk of bias in the included studies is presented in Figures 2, 3.

### 3.4 Overall effect of discharge planning on postpartum women’s health status

The meta-analysis included 14 studies involving 8,454 participants. Four health-related outcomes were analyzed: self and neonatal care competence, depression, anxiety, and breastfeeding self-efficacy. Subgroup analyses were conducted based on the presence or absence of theoretical frameworks and follow-up assessments.

#### 3.4.1 Depression

**Figure 4:**
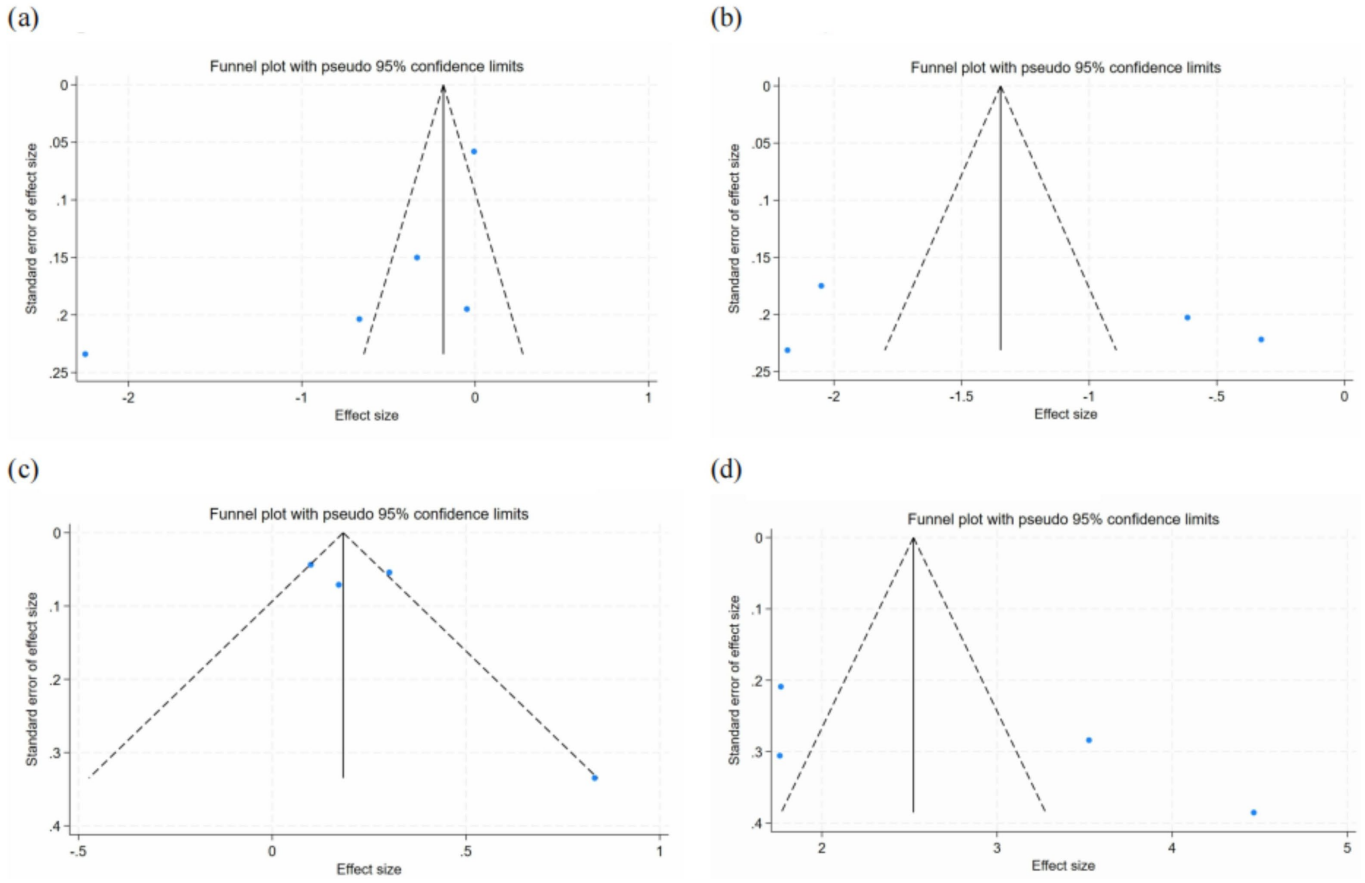
Funnel plots for the included studies assessing: **(a)** Depression; **(b)** Anxiety; **(c)** Self-Care and Neonatal Care Competence; and **(d)** Breastfeeding Self-Efficacy.

**Figure 5:**
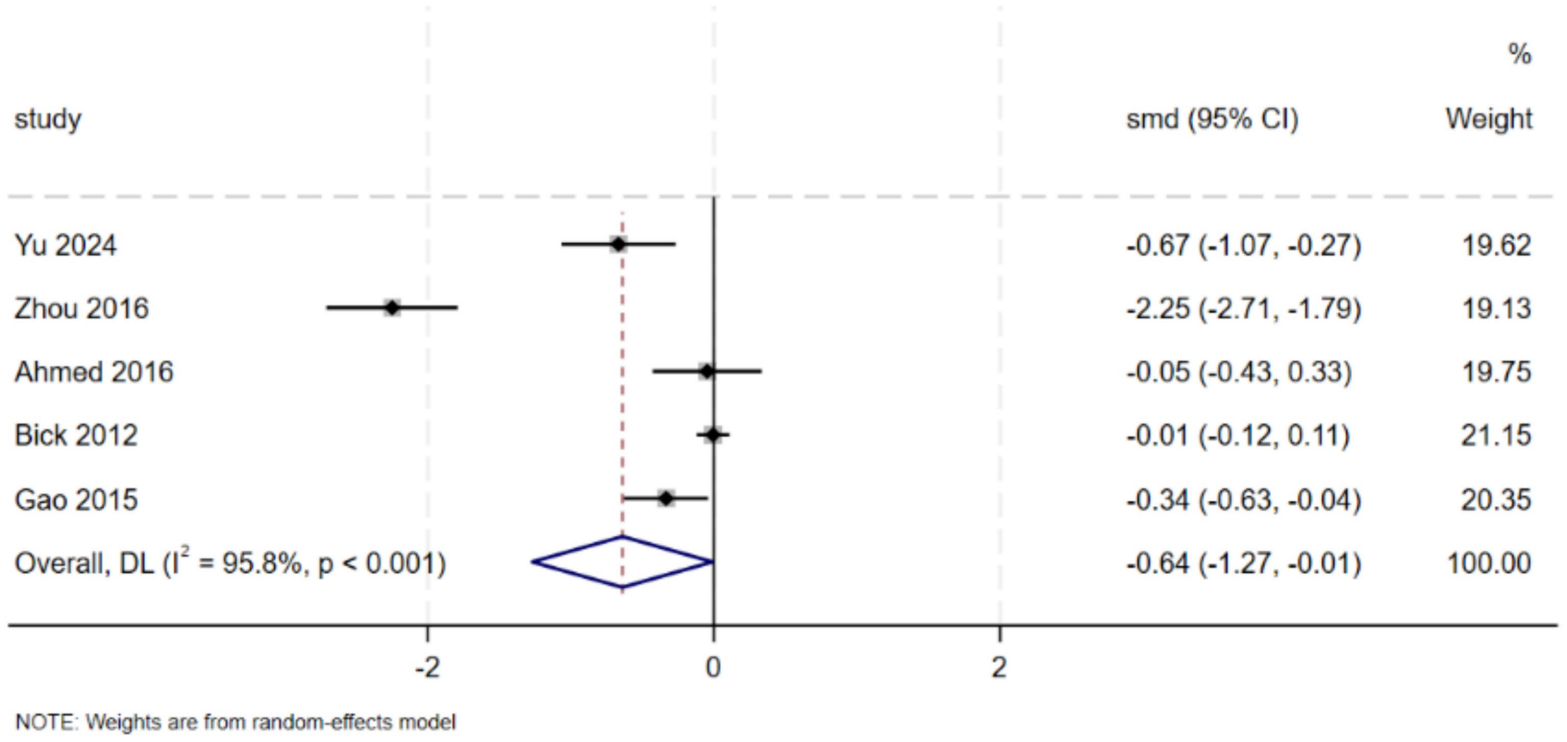
Effect of discharge planning on depression.

A total of 5 studies evaluated the effect of discharge planning interventions on depression (33, 34, 36, 37, 43). The results demonstrated that discharge planning interventions significantly reduced depressive symptoms in postpartum women (SMD = −0.64, 95% CI: [−1.27, −0.01], *p* = 0.046). Significant heterogeneity was observed among the studies (*I*^2^ = 95.8%, *p* < 0.001). Funnel plot asymmetry was noted (Figure 4a), but subsequent Egger’s test and Begg’s test revealed no significant publication bias (Egger’s test, *p* = 0.151; Begg’s test, *p* = 0.086). Meta-analysis is presented in Figure 5.

#### 3.4.2 Anxiety

**Figure 6:**
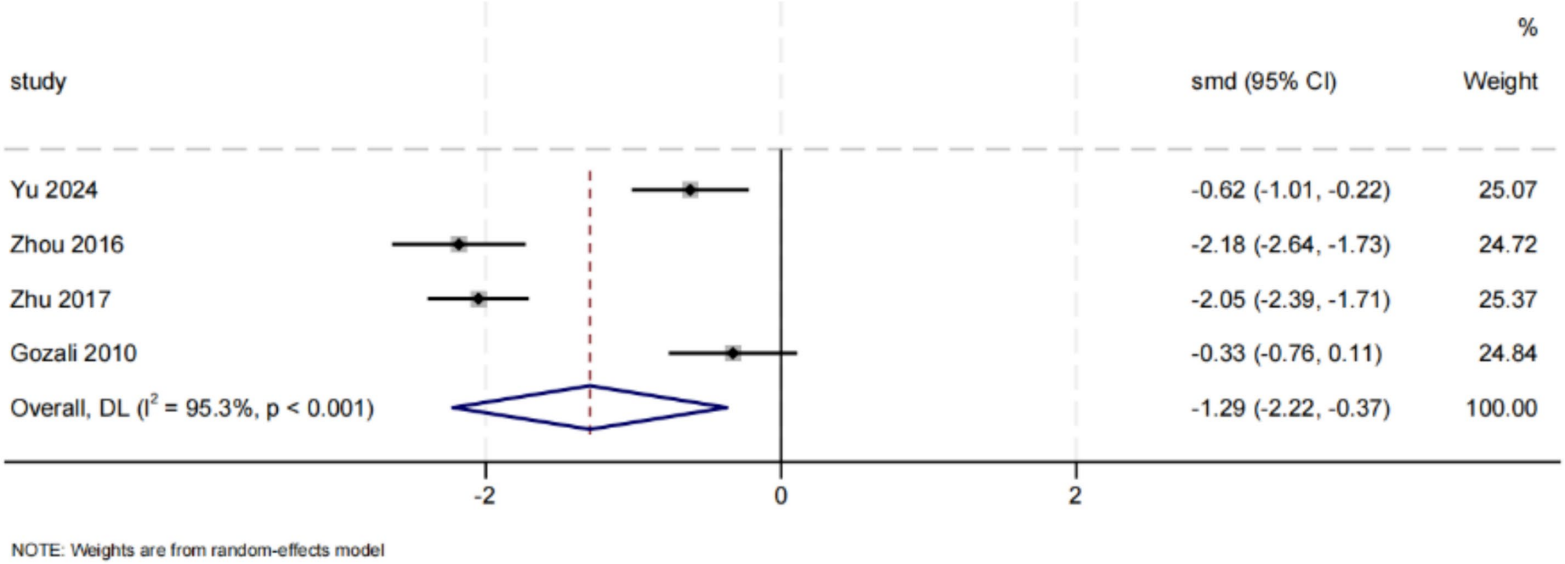
Effect of discharge planning on anxiety.

Four studies assessed the impact of discharge planning interventions on anxiety (33–35, 38). The results indicated that these interventions significantly alleviated anxiety in postpartum women (SMD = −1.29, 95% CI: [−2.22, −0.37], *p* = 0.006). Substantial heterogeneity was present across studies (*I*^2^ = 95.3%, *p* < 0.001). Although funnel plot asymmetry was observed (Figure 4b), neither Egger’s test nor Begg’s test detected significant publication bias (Egger’s test, *p* = 0.662; Begg’s test, *p* = 0.734). Meta-analysis is presented in Figure 6.

#### 3.4.3 Self-care and neonatal care competence

**Figure 7:**
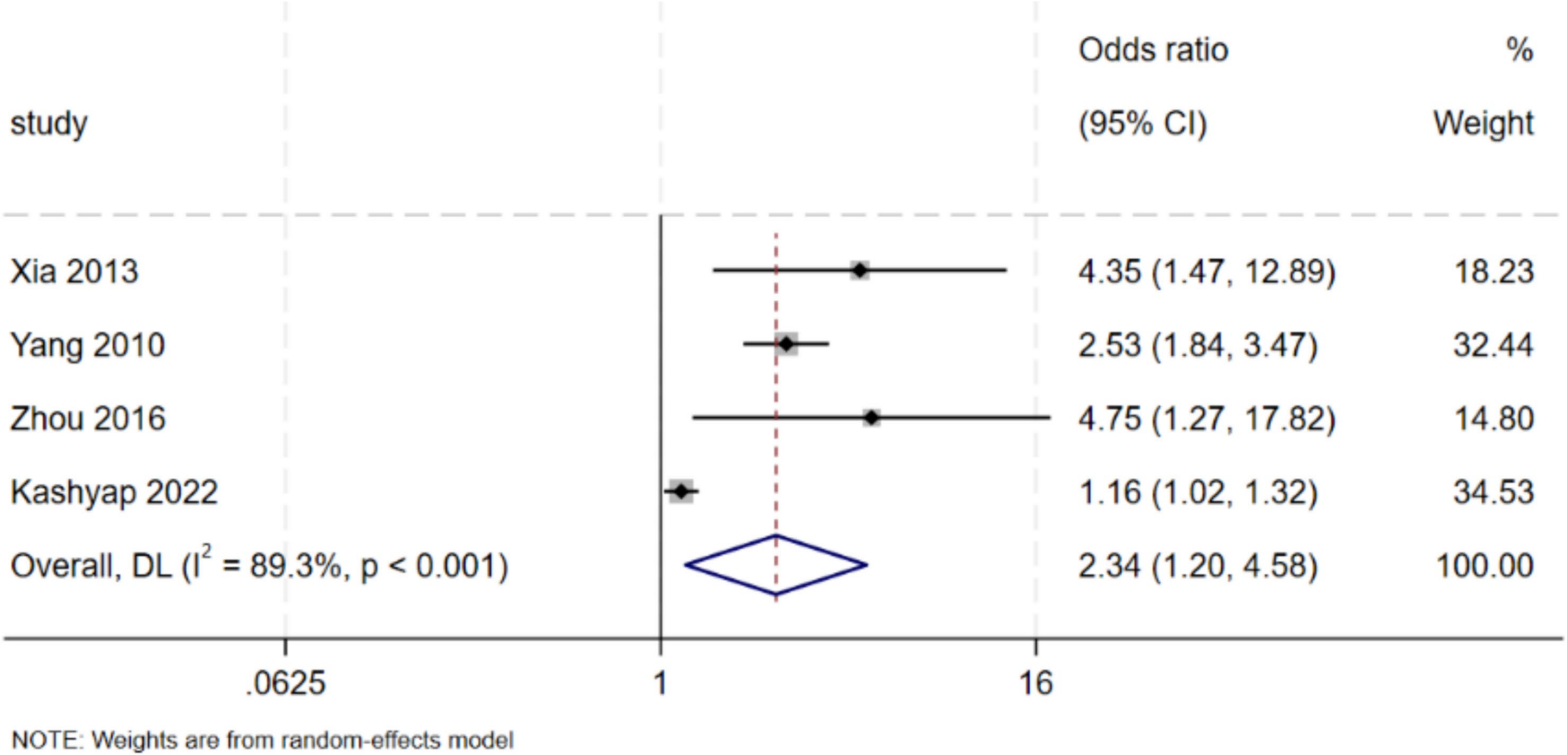
Effect of discharge planning on self-care and neonatal care competence.

Seven studies examined the effect of discharge planning interventions on self-care and neonatal care competence (30, 31, 34, 35, 38, 39, 41). Among these, 4 studies reported outcomes in dichotomous form and were included in the meta-analysis (30, 31, 34, 41). The results revealed that discharge planning interventions significantly improved maternal self-care and neonatal care competence (OR = 2.34, 95% CI: [1.20, 4.58], *p* = 0.013). High heterogeneity was observed (*I*^2^ = 89.3%, *p* < 0.001). Funnel plot asymmetry was noted (Figure 4c), but no significant publication bias was detected by Egger’s test (*p* = 0.161) or Begg’s test (*p* = 1.000). The remaining three studies, which were not included in the meta-analysis, consistently reported significant improvements in self-care and neonatal care competence following discharge planning interventions. Meta-analysis is presented in Figure 7.

#### 3.4.4 Breastfeeding self-efficacy

**Figure 8:**
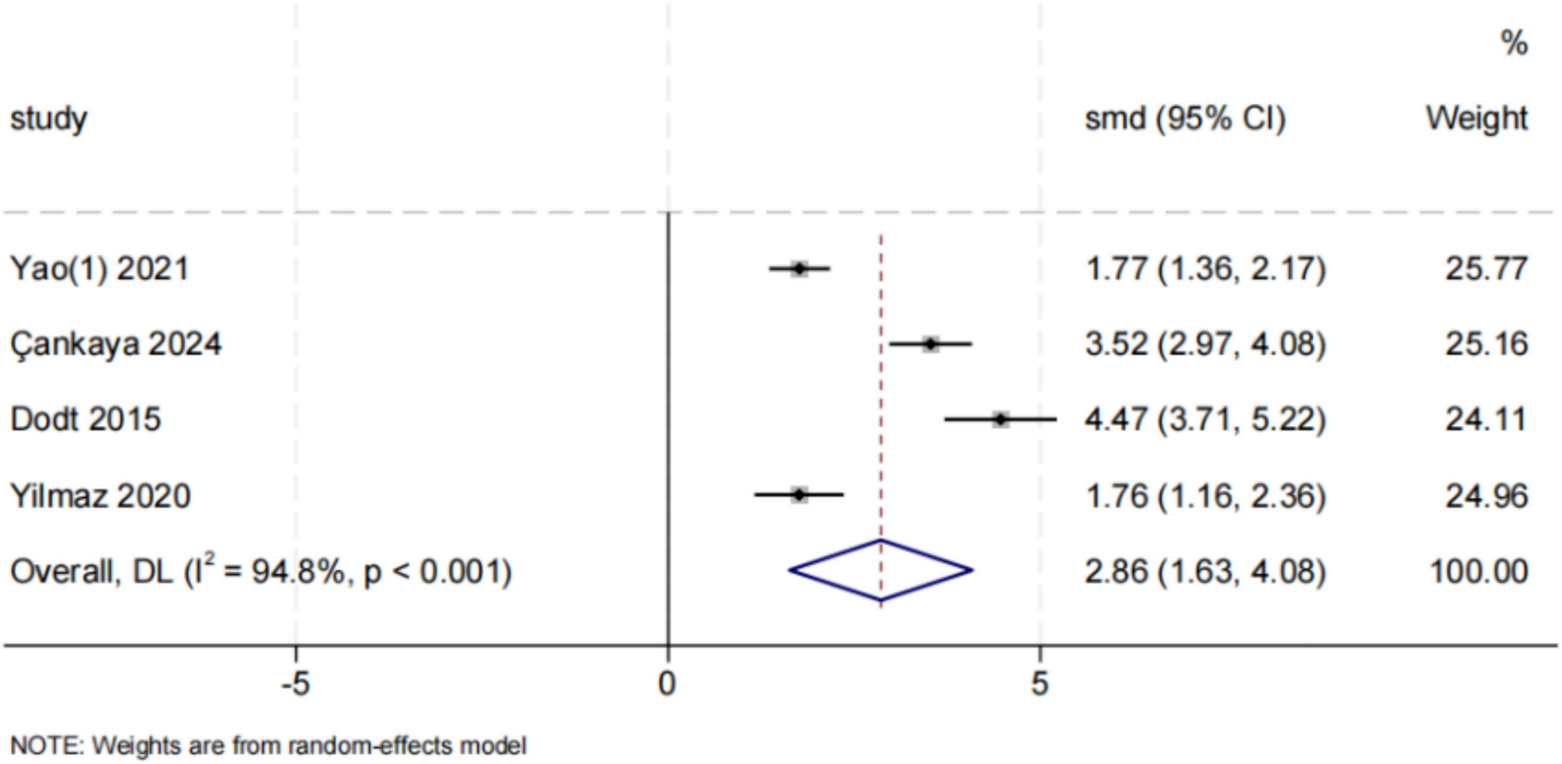
Effect of discharge planning on breastfeeding self-efficacy.

Four studies evaluated the influence of discharge planning interventions on breastfeeding self-efficacy (32, 39, 40, 42). The results demonstrated a significant enhancement in breastfeeding self-efficacy among postpartum women (SMD = 2.86, 95% CI: [1.63, 4.08], *p* < 0.001). Considerable heterogeneity was observed (*I*^2^ = 94.8%, *p* < 0.001). Although funnel plot asymmetry was present (Figure 4d), neither Egger’s test (*p* = 0.259) nor Begg’s test (*p* = 0.308) indicated significant publication bias. Meta-analysis is presented in Figure 8.

### 3.5 Sensitivity analysis

In the meta-analysis of four health-related outcomes, significant heterogeneity was observed across all indicators, suggesting substantial variability among the included studies. To further investigate the sources of heterogeneity, sensitivity analyses were performed by sequentially excluding individual studies to evaluate their impact on the overall effect size and heterogeneity. In the sensitivity analysis for depression, the exclusion of Zhou 2016 resulted in a significant reduction in heterogeneity (*I*^2^ = 76%, *p* = 0.005), indicating that this study might be a major contributor to the high heterog eneity. For self-care and neonatal care competency, the removal of Kashyap 2022 eliminated heterogeneity (*I*^2^ = 0%, *p* > 0.05), suggesting that this study was likely the primary source of heterogeneity. In contrast, no significant changes in heterogeneity were observed in the sensitivity analyses for anxiety and breastfeeding self-efficacy, implying that the heterogeneity in these outcomes may be associated with other factors.

### 3.6 Subgroup analysis

Subgroup analyses were conducted based on the theoretical framework of discharge planning interventions and follow-up status (Table 1). The results revealed no significant subgroup differences between studies with a theoretical framework and those without. However, among depression studies with a theoretical framework, no within-group heterogeneity was observed (SMD = −0.22, 95% CI: [−0.49, 0.06], *I*^2^ = 26.7%). Additionally, no significant subgroup differences were found between studies with follow-up and those without.

**Table 1:** Subgroup analysis on the impact of discharge planning on depression, anxiety, self-care and neonatal care competency, and breastfeeding self-efficacy.

Variables	Depression			Anxiety			Self-care and neonatal care competence			Breastfeeding self-efficacy		
	*K*	SMD (95 %CI)	*p*-value	*K*	SMD (95% CI)	*p*-value	*K*	OR (95 %CI)	*p*-value	*K*	SMD (95% CI)	*p*-value
Theoretical framework			0.256			0.944			0.065			0.772
Yes	2	−0.22 (−0.49, 0.06)		2	−1.34 (−2.74, 0.07)		1	1.10 (1.01, 1.20)		2	2.63 (1.63, 4.08)	
No	2	−0.96 (−2.21, −0.29)		2	−1.25 (−3.07, 0.57)		3	1.32 (1.12, 1.56)		2	3.10 (0.45, 5.75)	
Follow-up			0.496			0.863			0.060			0.780
Yes	2	−0.35 (−0.67, −0.03)		2	−1.39 (−2.93, 0.14)		3	1.21 (1.06, 1.38)		2	3.10 (0.45, 5.74)	
No	2	−1.12 (−3.31, 1.08)		2	−1.19 (−2.88, 0.50)		1	2.30 (1.19, 4.43)		2	2.65 (0.92, 4.38)	

K: number of studies; CI: confidence interval; OR: odds ratio. SMD: standardized mean difference.

## 4 Discussion

This study represents the first systematic review and meta-analysis to comprehensively evaluate the impact of discharge planning interventions on multidimensional health outcomes in postpartum women, including mental health, self-care and neonatal care competencies, and breastfeeding self-efficacy. The results demonstrate that systematic discharge planning interventions yield significant improvements in postpartum women’s mental health (depression: SMD = −0.64, 95% CI: [−1.27, −0.01]; anxiety: SMD = −1.29, 95% CI: [−2.22, −0.37]), self-care and neonatal care competencies (OR = 2.34, 95% CI: [1.20, 4.58]), and breastfeeding self-efficacy (SMD = 2.86, 95% CI: [1.63, 4.08]). These findings underscore the importance of discharge planning in promoting the overall health of postpartum women, particularly in terms of mental health and self-care capabilities. These findings highlight the critical role of discharge planning in promoting the overall health of postpartum women, particularly in terms of their mental wellbeing and self care capacity. The effectiveness observed is likely attributable to the integrated model commonly adopted in such interventions—wherein predischarge education (E) establishes a foundational knowledge base, while post discharge telephone follow up (T) and information exchange platforms (P) extend professional support into the community and home settings. This approach addresses the ongoing needs of postpartum women during the transition period and facilitates their sustained competence in self care, newborn care, breastfeeding, and other key domains.

### 4.1 Mechanisms underlying improvements in mental health outcomes

The study results indicate that discharge planning interventions significantly reduce depression and anxiety levels in postpartum women, which aligns with previous findings (44, 45). These studies collectively demonstrate that systematic discharge planning provides essential psychological support, further confirming its efficacy in improving postpartum mental health. Khamidullina et al. reported (2) that the occurrence rate of postpartum depression varies across populations, with higher rates observed in low- and middle-income countries. Systematic discharge planning can mitigate the impact of limited healthcare resources and inadequate social support by offering continuous psychological assistance and educational resources, thereby enhancing maternal psychological resilience (46, 47). Additionally, structured follow-up arrangements within discharge planning ensure sustained support after hospital discharge, reducing mental health complications associated with psychological stress.

Notably, high heterogeneity was observed for both depression (*I*^2^ = 95.8%, *p* < 0.001) and anxiety (*I*^2^ = 95.3%, *p* < 0.001), indicating substantial variability in intervention effects across studies. Subsequent sensitivity analysis revealed that the heterogeneity in depression was partially attributable to the study by Zhou (34). Specifically, Zhou employed a homogeneous sample consisting exclusively of caesarean section patients (aged 23–45 years), whereas other studies included more diverse populations with varying delivery methods, ethnicities, and geographic regions. In addition to differences in population characteristics and intervention components, methodological variability in outcome assessment may also have contributed to the heterogeneity. Although comparable instruments such as the EPDS and SDS were used, studies adopted different cut-off values to define clinically significant symptoms. Variations in threshold selection can meaningfully affect event rates and effect size estimates, particularly when outcomes are analyzed dichotomously, as lower cut-offs increase case identification whereas higher thresholds yield more conservative classifications. Such inconsistencies in outcome operationalization may partly explain the variability in reported intervention effects. For anxiety, no single study emerged as a dominant source of heterogeneity, suggesting that multiple factors—including differences in measurement tools, scoring thresholds, population profiles, and intervention intensity—likely acted in combination. Given the limited number of included studies, these findings should be interpreted cautiously. Greater standardization in measurement instruments and reporting of cut-off criteria would enhance comparability and strengthen future meta-analytic syntheses.

From an implementation perspective, discharge planning may function as a feasible approach to mitigate fragmentation in postpartum mental health care by promoting continuity between inpatient services and community-based support (48). Rather than relying on a single provider, many discharge planning interventions distribute responsibilities across nurses, obstetricians, and community health workers, which may partially address challenges associated with multidisciplinary coordination (49). However, the effectiveness of such approaches is likely influenced by local healthcare infrastructure, availability of trained personnel, and the degree of integration between hospital and community services. These contextual factors should be considered when interpreting the observed mental health benefits.

### 4.2 Self-care and neonatal care competency

Discharge planning interventions demonstrated significant efficacy in enhancing postpartum women’s self-care and neonatal care competencies. Through systematic predischarge assessments and individualized health education, parturients acquired improved postpartum care knowledge and skills, thereby bolstering their confidence in self-care and neonatal care practices (50). The study indicated (4) that postpartum women commonly face deficits in self-care and neonatal care knowledge and skills, whereas structured discharge planning effectively addresses these gaps through targeted education and support. Furthermore, the structured follow-up arrangements embedded in discharge planning ensured continuous post discharge support, mitigating health complications arising from inadequate care knowledge. It is important to note that the effectiveness of discharge planning in improving self-care and neonatal care competencies also depends on maternal engagement and adherence. Postpartum women often prioritize infant care over their own health needs, which may limit active participation in self-care–focused interventions. Interventions that integrate maternal self-care guidance into infant-centered routines, emphasize the interdependence between maternal wellbeing and infant outcomes, and provide flexible, low-burden support may be more acceptable and feasible in real-world settings (51). Discharge planning interventions not only improved maternal self-care capacity but also significantly enhanced proficiency in neonatal care skills (52). Dol et al. (10) further demonstrated that personalized neonatal care guidance provided through discharge planning facilitated better skill acquisition among mothers, thereby improving neonatal health outcomes. These findings underscore the pivotal role of discharge planning in promoting postpartum self-care and neonatal care competency while highlighting its substantial impact on improving maternal overall health.

Due to high heterogeneity (*I*^2^ = 89.3%, *p* < 0.001), sensitivity analysis was performed, revealing that the exclusion of Kashyap et al. eliminated heterogeneity (*I*^2^ = 0%, *p* > 0.05) (41), suggesting this study as the primary source of heterogeneity. Two key factors were identified: First, Kashyap et al. employed a quasi-experimental design, which, despite controlling for some confounders, possesses a lower evidence level than RCTs and is more susceptible to external influences. In contrast, all other included studies were RCTs, which minimized potential confounders through randomization, yielding more robust evidence. Second, Kashyap et al. featured a larger sample size, encompassing participants from 11 district hospitals across two Indian states, thereby covering a broader demographic and geographic scope. However, outcome assessment relied predominantly on telephone surveys, with non-responsive families excluded, potentially introducing selection bias. Conversely, other studies, despite smaller sample sizes, utilized diversified evaluation methods—including scale scoring, questionnaires, and skill demonstrations—providing a more comprehensive assessment of intervention efficacy.

### 4.3 Breastfeeding self-efficacy

The study results demonstrated that discharge planning interventions significantly improved breastfeeding self-efficacy among postpartum women. Breastfeeding is not only critical for neonatal health but also closely associated with maternal postpartum recovery (53). By providing breastfeeding education and support, discharge planning enhances maternal confidence in breastfeeding and increases breastfeeding success rates (54). Symonds et al. (55) highlighted that in low- and middle-income countries, significant gaps exist in the accessibility and quality of postpartum care, and discharge planning can effectively address these disparities through sustained support. Furthermore, discharge planning offers personalized breastfeeding guidance, helping mothers overcome challenges encountered during breastfeeding, thereby improving breastfeeding continuity. The positive impact of discharge planning on breastfeeding self-efficacy may extend beyond the immediate postpartum period, although existing evidence is primarily based on short-term follow-up and should be interpreted with caution. The study reported (56, 57) that discharge planning, by providing continuous breastfeeding support, significantly enhanced maternal breastfeeding self-efficacy within 4 months postpartum. These findings underscore the pivotal role of discharge planning in promoting breastfeeding self-efficacy among postpartum women.

### 4.4 The role of theoretical frameworks and follow-up in discharge planning interventions

In the subgroup analysis of this study, although no significant subgroup differences were observed between studies with and without theoretical support, studies with a theoretical foundation for depression interventions exhibited no within-group heterogeneity (SMD = −0.2, 95% CI: [−0.48, 0.08], *I*^2^ = 37.8%, *p* = 0.206). This suggests that theoretical frameworks may provide guidance in specific health outcomes (e.g., depression), enhancing intervention consistency and effectiveness. At the same time, the overall lack of significant subgroup differences may be attributed to the diversity of theoretical frameworks, limitations in study design and implementation, and the limited number of included studies. Similarly, subgroup analysis did not reveal significant differences between studies with and without follow-up. Nonetheless, follow-up remains potentially important in discharge planning interventions, with its impact likely varying depending on intervention specifics, implementation methods, and study population characteristics. In addition, the practical application of theoretical frameworks and follow-up strategies may be constrained by resource availability, workforce capacity, and organizational support within healthcare systems, which may partly explain the inconsistent effects observed across studies. Accordingly, Future research should further investigate the mechanisms of theoretical frameworks and follow-up across different health outcomes and populations, clarify the selection and application of theoretical frameworks, optimize follow-up design, and increase the number and diversity of studies to more accurately assess their roles and refine the design and implementation of discharge planning interventions.

### 4.5 Strengths and limitations

In this meta-analysis, both RCTs and quasi-experimental studies were included to synthesize a more comprehensive evidence base. While RCTs remain the gold standard for internal validity, the implementation of strict individual randomization in postpartum discharge planning is frequently precluded by pragmatic and ethical constraints. Specifically, the risk of ‘treatment diffusion’ or ‘information contamination’ is inherently high within obstetric environments. Because discharge planning involves intensive health education and behavioral interventions, participants within the same ward or social network often share educational materials and care advice, which can artificially dilute the observable effect size of the intervention. Furthermore, in certain healthcare contexts, withholding structured discharge support from eligible postpartum women may pose ethical dilemmas, particularly when such interventions are integrated into institutional quality improvement mandates. Consequently, quasi-experimental designs are vital for evaluating the effectiveness of programs under real-world conditions. While these designs may be more susceptible to residual confounding compared to RCTs, they offer a more pragmatic reflection of routine healthcare delivery and enhance the external validity of our findings.

Compared with previous studies, our research exhibits several notable strengths. First, by incorporating a broader range of studies and employing rigorous meta-analysis techniques, our study provides a more comprehensive and up-to-date synthesis of evidence. Second, the included studies encompassed diverse populations and healthcare settings, which enhances the contextual breadth of the findings, although the applicability of results may vary across settings. Third, our study systematically evaluated multiple health-related outcomes, offering a more holistic understanding of the impact of discharge planning on postpartum women’s health. However, several limitations of our study should be acknowledged. A key limitation is the heterogeneity among the included studies, which may affect the precision of the pooled estimates. In addition, the relatively small number of studies and limited sample sizes in certain subgroups may have reduced statistical power, and some individual studies reported non-significant results, warranting cautious interpretation of the findings. Variability in study quality may further influence the robustness of the conclusions. Additionally, only two included studies reported neonatal or infant complications, which limited the ability to draw firm conclusions regarding the effects of discharge planning on neonatal outcomes. Future studies should prioritize evaluating these outcomes with standardized definitions.

To address these limitations, future research should focus on the following aspects. First, more high-quality randomized controlled trials with larger sample sizes are needed to provide stronger evidence regarding the efficacy of discharge planning interventions. Second, future studies should investigate diverse populations and settings, including low- and middle-income countries, to ensure broader applicability of the findings. Third, further research should explore the long-term effects of discharge planning interventions on maternal and infant health outcomes, as most existing studies have focused on short-term outcomes. Finally, cost-effectiveness analyses of different discharge planning interventions should be conducted to inform policy and practice decisions.

## 5 Conclusion

This systematic review and meta-analysis demonstrates that discharge planning significantly improves mental health, physiological indicators, and maternal-neonatal self-care among postpartum women. Future trials should address current methodological limitations and assess long-term outcomes to refine evidence-based discharge strategies that promote maternal and infant wellbeing.

## Data Availability

The following information was supplied regarding data availability: This is a systematic review/meta-analysis.

## References

[1] Saharoy R Potdukhe A Wanjari M Taksande AB. Postpartum depression and maternal care: exploring the complex effects on mothers and infants. Cureus. (2023) 15:e41381. doi: 10.7759/cureus.41381, 37546054 PMC10400812

[2] Khamidullina Z Marat A Muratbekova S Mustapayeva NM Chingayeva GN Shepetov AM . Postpartum depression epidemiology, risk factors, diagnosis, and management: an appraisal of the current knowledge and future perspectives. J Clin Med. (2025) 14:2418. doi: 10.3390/jcm14072418, 40217868 PMC11989329

[3] Vogel JP Jung J Lavin T Simpson G Kluwgant D Abalos E . Neglected medium-term and long-term consequences of labour and childbirth: a systematic analysis of the burden, recommended practices, and a way forward. Lancet Glob Health. (2024) 12:e317–30. doi: 10.1016/s2214-109x(23)00454-0, 38070535 PMC10805007

[4] Nguyen PY Caddy C Wilson AN Blackburn K Page MJ Gülmezoglu AM . Self-care interventions for preconception, antenatal, intrapartum and postpartum care: a scoping review. BMJ Open. (2023) 13:e068713. doi: 10.1136/bmjopen-2022-068713, 37164476 PMC10173967

[5] Golnam M Hassani L Goodarzi RS Ghanbarnejad A. The effectiveness of a theory-based health education program on self-efficacy and breastfeeding behaviors continuity of working mothers in Iran. Sci Rep. (2025) 15:5625. doi: 10.1038/s41598-025-89943-9, 39955380 PMC11830010

[6] Green JE Ang N Harris-Roxas B Baird K Roth H Henry A. Exploring Australian knowledge and practice for maternal postnatal transition of care between hospital and primary care: a scoping review. Women Birth. (2025) 38:101852. doi: 10.1016/j.wombi.2024.101852, 39752774

[7] Adams YJ Miller ML Agbenyo JS Ehla EE Clinton GA. Postpartum care needs assessment: women's understanding of postpartum care, practices, barriers, and educational needs. BMC Pregnancy Childbirth. (2023) 23:502. doi: 10.1186/s12884-023-05813-0, 37420215 PMC10327352

[8] McCarter D Law AA Cabullo H Pinto K. Scoping review of postpartum discharge education provided by nurses. J Obstet Gynecol Neonatal Nurs. (2022) 51:377–87. doi: 10.1016/j.jogn.2022.03.002, 35483423 PMC9257451

[9] Smith H Harvey C Portela A. Discharge preparation and readiness after birth: a scoping review of global policies, guidelines and literature. BMC Pregnancy Childbirth. (2022) 22:281. doi: 10.1186/s12884-022-04577-3, 35382773 PMC8985304

[10] Dol J Hughes B Bonet M Dorey R Dorling J Grant A . Timing of maternal mortality and severe morbidity during the postpartum period: a systematic review. JBI Evid Synth. (2022) 20:2119–94. doi: 10.11124/jbies-20-00578, 35916004 PMC9594153

[11] Madray C Richardson J Hornsby P Grello C Drake E Kellams A. Exploring the unmet needs of postpartum mothers: a qualitative study. J Perinat Educ. (2022) 31:71–81. doi: 10.1891/jpe-2021-00009, 35386495 PMC8970134

[12] Braet A Weltens C Vleugels A. Effectiveness of discharge interventions from hospital to home to reduce readmissions: a systematic review. JBI Libr Syst Rev. (2012) 10:1–13. doi: 10.11124/jbisrir-2012-310, 27820399

[13] Khademi K Kaveh MH. Social support as a coping resource for psychosocial conditions in postpartum period: a systematic review and logic framework. BMC Psychol. (2024) 12:301. doi: 10.1186/s40359-024-01814-6, 38807228 PMC11131291

[14] Tani F Castagna V. Maternal social support, quality of birth experience, and post-partum depression in primiparous women. J Matern Fetal Neonatal Med. (2017) 30:689–92. doi: 10.1080/14767058.2016.1182980, 27123699

[15] Wang Z Zhang Q Nie R Zhou L Zhao C Cao Y . Knowledge attitude and practice of pregnant women on postnatal depression in Henan Province China. Sci Rep. (2025) 15:24810. doi: 10.1038/s41598-025-08274-x, 40640223 PMC12246150

[16] Shang J Dolikun N Tao X Zhang P Woodward M Hackett ML . The effectiveness of postpartum interventions aimed at improving women's mental health after medical complications of pregnancy: a systematic review and meta-analysis. BMC Pregnancy Childbirth. (2022) 22:809. doi: 10.1186/s12884-022-05084-1, 36329395 PMC9632104

[17] Collaborators GFaF. Global fertility in 204 countries and territories, 1950-2021, with forecasts to 2100: a comprehensive demographic analysis for the global burden of disease study 2021. Lancet. (2024) 403:2057–99. doi: 10.1016/s0140-6736(24)00550-6, 38521087 PMC11122687

[18] Cresswell JA Alexander M Chong MYC Link HM Pejchinovska M Gazeley U . Global and regional causes of maternal deaths 2009-20: a WHO systematic analysis. Lancet Glob Health. (2025) 13:e626–34. doi: 10.1016/s2214-109x(24)00560-6, 40064189 PMC11946934

[19] Cumpston MS McKenzie JE Welch VA Brennan SE. Strengthening systematic reviews in public health: guidance in the Cochrane handbook for systematic reviews of interventions, 2nd edition. J Public Health. (2022) 44:e588–92. doi: 10.1093/pubmed/fdac036, 35352103 PMC9715291

[20] Page MJ McKenzie JE Bossuyt PM Boutron I Hoffmann TC Mulrow CD . The PRISMA 2020 statement: an updated guideline for reporting systematic reviews. BMJ (Clinical Research Ed). (2021) 372:n71. doi: 10.1136/bmj.n71, 33782057 PMC8005924

[21] Sterne JAC Savović J Page MJ Elbers RG Blencowe NS Boutron I . RoB 2: a revised tool for assessing risk of bias in randomised trials. BMJ (Clinical Research Ed.). (2019) 366:l4898. doi: 10.1136/bmj.l4898 31462531

[22] Sterne JA Hernán MA Reeves BC Savović J Berkman ND Viswanathan M . ROBINS-I: a tool for assessing risk of bias in non-randomised studies of interventions. BMJ (Clinical Research Ed). (2016) 355:i4919. doi: 10.1136/bmj.i4919 PMC5062054 27733354

[23] Wang X Zhou Y Zhou Y. Effect of enhanced perioperative health education on breastfeeding in cesarean section. J Med Inf. (2007) 5:831–2. doi: 10.3969/j.issn.1006-1959.2007.05.060

[24] Xie A Fan J Tang Y. Application of clinical nursing path in the health education of elective caesarean section. China Medical Herald. (2010) 7:74–5. doi: 10.3969/j.issn.1673-7210.2010.17.038

[25] Yao L Yu Y He Y Sheng J. The influence of health education based on the behavioral change model on postpartum nutritional intake, milk secretion and breastfeeding behavior of mothers. Matern Child Health Care China. (2021) 36:4835–8. doi: 10.19829/j.zgfybj.issn.1001-4411.2021.20.062

[26] Consales A Colombo L Zanotta L Morniroli D Sannino P Rampini S . Pilot feasibility study of a hospital-based post-Natal educational intervention on new mothers in a BFHI-compliant tertiary referral Center for Neonatal Care. Int J Environ Res Public Health. (2022) 19:2020. doi: 10.3390/ijerph19042020, 35206209 PMC8871806

[27] Estalella I San Millán J Trincado MJ Maquibar A Martínez-Indart L San SM. Evaluation of an intervention supporting breastfeeding among late-preterm infants during in-hospital stay. Women Birth. (2020) 33:e33–8. doi: 10.1016/j.wombi.2018.11.003, 30527733

[28] Fu IC Fong DY Heys M Lee IL Sham A Tarrant M. Professional breastfeeding support for first-time mothers: a multicentre cluster randomised controlled trial. BJOG. (2014) 121:1673–83. doi: 10.1111/1471-0528.12884, 24861802

[29] Johnston JS Suri P Yan S Chandrasekar A Singla S Ward VC . A mobile messaging service for families on postnatal knowledge and practices: a cluster randomized trial, India. Bull World Health Organ. (2025) 103:255–65. doi: 10.2471/blt.24.292145 40207245 PMC11978415

[30] Xia Q Sun J. Application of professional skills guidance table in health education after cesarean section. Chin Nurs Res. (2013) 27:921–2. doi: 10.3969/j.issn.1009-6493.2013.10.026

[31] Yang J Li Y. The application of goal-based health education in pregnant women. Hainanme Dical Journal. (2010) 21:153–5. doi: 10.3969/j.issn.1003-6350.2010.15.067

[32] Yao L Han P He Y. Effect of feeding education based on the behavior change model on breastfeeding confidence and feeding behaviors in primiparous women. Matern Child Health Care China. (2021) 36:5806–9. doi: 10.19829/j.zgfybj.issn.1001-4411.2021.24.060

[33] Yu Y Du Y Fang F. The influence of a personalized health guidance program based on psychological regulation on the treatment compliance of postpartum psychological state and pelvic floor function of parturients. Matern Child Health Care China. (2024) 39:535–8. doi: 10.19829/j.zgfybj.issn.1001-4411.2024.03.037

[34] Zhou Y. Evaluation on the clinical effect of staged health education during perinatal period of cesarean section. China Contin Med Educ. (2016) 8:181–3. doi: 10.3969/j.issn.1674-9308.2016.36.102

[35] Zhu H. The application of collaborative care in mother-baby rooming-together setup. Womens Health Res. (2017) 19:91. doi: 10.3969/j.issn.2096-0417.2017.19.064

[36] Gao LL Xie W Yang X Chan SW. Effects of an interpersonal-psychotherapy-oriented postnatal programme for Chinese first-time mothers: a randomized controlled trial. Int J Nurs Stud. (2015) 52:22–9. doi: 10.1016/j.ijnurstu.2014.06.006, 24994573

[37] Ahmed AH Roumani AM Szucs K Zhang L King D. The effect of interactive web-based monitoring on breastfeeding exclusivity, intensity, and duration in healthy, term infants after hospital discharge. J Obstet Gynecol Neonatal Nurs. (2016) 45:143–54. doi: 10.1016/j.jogn.2015.12.001, 26779838 PMC4789120

[38] Gozali A Gibson S Lipton LR Pressman AW Hammond BS Dumitriu D. Assessing the effectiveness of a pediatrician-led newborn parenting class on maternal newborn-care knowledge, confidence and anxiety: a quasi-randomized controlled trial. Early Hum Dev. (2020) 147:105082. doi: 10.1016/j.earlhumdev.2020.105082, 32512498

[39] Çankaya S Tezgören E Dikmen HA. The effects of the intrapartum care model given in line with the recommendations of the World Health Organization (WHO) on the mother's maternal behavior towards her baby, breastfeeding self-efficacy, breastfeeding success, and hospital discharge readiness: a randomized controlled trial. Arch Gynecol Obstet. (2024) 310:3009–27. doi: 10.1007/s00404-024-07844-0, 39601811

[40] Yilmaz F Küçükoğlu S Aytekin Özdemir A Oğul T Aşki N. The effect of kangaroo mother care, provided in the early postpartum period, on the breastfeeding self-efficacy level of mothers and the perceived insufficient milk supply. J Perinat Neonatal Nurs. (2020) 34:80–7. doi: 10.1097/jpn.0000000000000434, 31895303

[41] Kashyap S Spielman AF Ramnarayan N Sd S Pant R Kaur B . Impact of family-centred postnatal training on maternal and neonatal health and care practices in district hospitals in two states in India: a pre-post study. BMJ Open Qual. (2022) 11:e001462. doi: 10.1136/bmjoq-2021-001462, 35545272 PMC9092167

[42] Dodt RC Joventino ES Aquino PS Almeida PC Ximenes LB. An experimental study of an educational intervention to promote maternal self-efficacy in breastfeeding. Rev Lat Am Enfermagem. (2015) 23:725–32. doi: 10.1590/0104-1169.0295.2609, 26444176 PMC4623736

[43] Bick D Murrells T Weavers A Rose V Wray J Beake S. Revising acute care systems and processes to improve breastfeeding and maternal postnatal health: a pre and post intervention study in one English maternity unit. BMC Pregnancy Childbirth. (2012) 12:41. doi: 10.1186/1471-2393-12-41, 22672354 PMC3489602

[44] McCarter-Spaulding D Shea S. Effectiveness of discharge education on postpartum depression. MCN Am J Matern Child Nurs. (2016) 41:168–72. doi: 10.1097/nmc.0000000000000236, 27128643 PMC4852281

[45] Dennis CL. Psychosocial and psychological interventions for prevention of postnatal depression: systematic review. BMJ. (2005) 331:15. doi: 10.1136/bmj.331.7507.15, 15994688 PMC558531

[46] Dharni N Essex H Bryant MJ de Cronin Chavez A Willan K Farrar D . The key components of a successful model of midwifery-led continuity of carer, without continuity at birth: findings from a qualitative implementation evaluation. BMC Pregnancy Childbirth. (2021) 21:205. doi: 10.1186/s12884-021-03671-2, 33711957 PMC7955626

[47] Myors KA Schmied V Johnson M Cleary M. Collaboration and integrated services for perinatal mental health: an integrative review. Child Adolesc Mental Health. (2013) 18:1–10. doi: 10.1111/j.1475-3588.2011.00639.x, 32847263

[48] Tyler N Hodkinson A Planner C Angelakis I Keyworth C Hall A . Transitional care interventions from hospital to community to reduce health care use and improve patient outcomes: a systematic review and network Meta-analysis. JAMA Netw Open. (2023) 6:e2344825. doi: 10.1001/jamanetworkopen.2023.44825, 38032642 PMC10690480

[49] Brown CL Tittlemier BJ Tiwari KK Loewen H. Interprofessional teams supporting care transitions from hospital to community: a scoping review. Int J Integr Care. (2024) 24:1. doi: 10.5334/ijic.7623, 38618048 PMC11012160

[50] Mokhtari F Bahadoran P Baghersad Z. Effectiveness of postpartum homecare program as a new method on mothers' knowledge about the health of the mother and the infant. Iran J Nurs Midwifery Res. (2018) 23:316–21. doi: 10.4103/Ijnmr.ijnmr_48_17, 30034494 PMC6034528

[51] Chivers BR Garad RM Moran LJ Lim S Harrison CL. Support seeking in the postpartum period: content analysis of posts in web-based parenting discussion groups. J Med Internet Res. (2021) 23:e26600. doi: 10.2196/26600, 34264198 PMC8323017

[52] Maluni J Oluoch D Molyneux S Boga M Jones C Murila F . After neonatal care, what next? A qualitative study of mothers' post-discharge experiences after premature birth in Kenya. Int J Equity Health. (2025) 24:17. doi: 10.1186/s12939-024-02340-y, 39833805 PMC11744954

[53] D'Hollander CJ McCredie VA Uleryk EM Keown-Stoneman CDG Birken CS O'Connor DL . Breastfeeding support provided by lactation consultants in high-income countries for improved breastfeeding rates, self-efficacy, and infant growth: a systematic review and meta-analysis protocol. Syst Rev. (2023) 12:75. doi: 10.1186/s13643-023-02239-9 37131212 PMC10152596

[54] Tseng JF Chen SR Au HK Chipojola R Lee GT Lee PH . Effectiveness of an integrated breastfeeding education program to improve self-efficacy and exclusive breastfeeding rate: a single-blind, randomised controlled study. Int J Nurs Stud. (2020) 111:103770. doi: 10.1016/j.ijnurstu.2020.103770, 32961461

[55] Symonds NE Vidler M Wiens MO Omar S English LL Ukah UV . Risk factors for postpartum maternal mortality and hospital readmission in low- and middle-income countries: a systematic review. BMC Pregnancy Childbirth. (2023) 23:303. doi: 10.1186/s12884-023-05459-y, 37120529 PMC10148415

[56] Chmelova K Berrington J Shenker N Zalewski S Rankin J Embleton N. Exploring human Milk, nutrition, growth, and breastfeeding rates at discharge(HUMMINGBIRD study): a protocol for a pilot randomised controlled trial. BMJ Paediatrics Open. (2023) 7:e001803. doi: 10.1136/bmjpo-2022-001803, 36882232 PMC10008155

[57] Hu L Ding T Hu J Luo B. Promoting breastfeeding in Chinese women undergoing cesarean section based on the health belief model: a randomized controlled trial. Medicine. (2020) 99:e20815. doi: 10.1097/md.0000000000020815, 32664074 PMC7360307

