# Supplementary material for: Effectiveness of discharge planning interventions on health-related outcomes among postpartum women: a systematic review and meta-analysis
Source: Front Public Health. 2026 Mar 23;14:1733799. doi: 10.3389/fpubh.2026.1733799 (PMC13051705; doi:10.3389/fpubh.2026.1733799)
Supplement: Supplementary file 1 [file Table_1.docx]

**Effectiveness of Discharge Planning Interventions on Health-related Outcomes Among Postpartum Women: A Systematic Review and Meta-analysis**

**Supplementary File S1**

The search strategies used for PubMed, Web of Science, PsycINFO, CINAHL, EMBASE, Cochrane Library, CNKI, Wanfang, and SinoMed databases.

**PubMed(784)**

| # | Search | Result |
| --- | --- | --- |
| 1 | (((((postpartum period[MeSH Terms]) OR (postpartum period[Title/Abstract])) OR (postnatal[Title/Abstract])) OR (postpartum[Title/Abstract])) OR (postpartum women[Title/Abstract])) OR (puerperium[Title/Abstract]) | [270,593](https://pubmed.ncbi.nlm.nih.gov/?term=(((((postpartum+period%5bMeSH+Terms%5d)+OR+(postpartum+period%5bTitle/Abstract%5d))+OR+(postnatal%5bTitle/Abstract%5d))+OR+(postpartum%5bTitle/Abstract%5d))+OR+(postpartum+women%5bTitle/Abstract%5d))+OR+(puerperium%5bTitle/Abstract%5d)&sort=relevance&show_snippets=off) |
| 2 | ((((((patient discharge[MeSH Terms]) OR (Discharge Planning[Title/Abstract])) OR (Discharge Plannings[Title/Abstract])) OR (patient discharge[Title/Abstract])) OR (hospital discharge[Title/Abstract])) OR (Discharge instructions[Title/Abstract])) OR (Discharge education[Title/Abstract]) | [77,708](https://pubmed.ncbi.nlm.nih.gov/?term=((((((patient+discharge%5bMeSH+Terms%5d)+OR+(Discharge+Planning%5bTitle/Abstract%5d))+OR+(Discharge+Plannings%5bTitle/Abstract%5d))+OR+(patient+discharge%5bTitle/Abstract%5d))+OR+(hospital+discharge%5bTitle/Abstract%5d))+OR+(Discharge+instructions%5bTitle/Abstract%5d))+OR+(Discharge+education%5bTitle/Abstract%5d)&sort=relevance&show_snippets=off) |
| 3 | ((((((((("mental health"[MeSH Terms])) OR ("Depression, Postpartum"[MeSH Terms])) OR ("breast feeding"[MeSH Terms])) OR ("infant health"[MeSH Terms])) OR ("Patient Satisfaction"[MeSH Terms])) OR ("quality of life"[MeSH Terms])) OR ("Self Care"[MeSH Terms])) OR ("Self-Management"[MeSH Terms])) OR ("patient readmission"[MeSH Terms]) | [604,985](https://pubmed.ncbi.nlm.nih.gov/?term=((((((((() |
| 4 | (((((((((((((((((((((((((((((((("postpartum recovery"[Title/Abstract]) OR ("postpartum complications"[Title/Abstract])) OR ("infection"[Title/Abstract])) OR ("mental health"[Title/Abstract])) OR ("Mental Hygiene"[Title/Abstract])) OR ("Depression, Postpartum"[Title/Abstract])) OR ("postnatal depression"[Title/Abstract])) OR ("depression"[Title/Abstract])) OR ("anxiety"[Title/Abstract])) OR ("breast feeding"[Title/Abstract])) OR ("Breastfeeding"[Title/Abstract])) OR ("infant health"[Title/Abstract])) OR ("Baby Health"[Title/Abstract])) OR ("Newborn Health"[Title/Abstract])) OR ("Neonatal Health"[Title/Abstract])) OR ("patient satisfaction"[Title/Abstract])) OR ("satisfaction"[Title/Abstract])) OR ("health services utilization"[Title/Abstract])) OR ("health care utilization"[Title/Abstract])) OR ("unmet needs"[Title/Abstract])) OR ("Life Quality"[Title/Abstract])) OR ("Health-Related Quality Of Life"[Title/Abstract])) OR ("HRQOL"[Title/Abstract])) OR ("quality of life"[Title/Abstract])) OR ("Self Management"[Title/Abstract])) OR ("Self-Management"[Title/Abstract])) OR ("Self Care"[Title/Abstract])) OR ("Self-Care"[Title/Abstract])) OR ("adverse outcome"[Title/Abstract])) OR ("adverse event"[Title/Abstract])) OR ("Rehospitalization"[Title/Abstract])) OR ("patient readmission"[Title/Abstract])) OR ("readmission"[Title/Abstract]) | [3,049,665](https://pubmed.ncbi.nlm.nih.gov/?term=(((((((((((((((((((((((((((((((() |
| 5 | #3 AND #4 | [3,223,750](https://pubmed.ncbi.nlm.nih.gov/?term=longquery7e99d049e4abae940c0f&sort=relevance&show_snippets=off) |
| 6 | #1 AND #2 AND #5 | 829 |
| 7 | English[Language] |  |
| 8 | #6 AND #7 | 784 |

**CINAHL(509)**

| # | Search | Result |
| --- | --- | --- |
| 1 | MH ( "Puerperium" OR "Postnatal Period") OR XB (postpartum period OR postnatal OR postpartum OR postpartum women OR puerperium) | 54,661 |
| 2 | MH ("Patient Discharge" OR "Patient Discharge Education" OR "Discharge Planning") OR XB (Patient Discharge OR Discharge Planning OR hospital discharge OR Discharge instructions OR Patient Discharge Education) | 53,288 |
| 3 | MH ("Mental Health" OR "Depression, Postpartum" OR "Breast Feeding" OR "Patient Satisfaction" OR "Quality of Life" OR "Self Care" OR "Self-Management" OR "Readmission") OR XB ("postpartum recovery" OR "postpartum complications" OR "infection" OR "mental health" OR "Mental Hygiene" OR "postpartum depression" OR "Depression, Postpartum" OR "depression" OR "anxiety" OR "breast feeding" OR Breastfeeding OR "infant health" OR "Baby Health" OR "Newborn Health" OR "patient satisfaction" OR satisfaction OR "health services utilization" OR "health care utilization" OR “unmet needs” OR "Life Quality" OR "Health-Related Quality Of Life" OR "HRQOL" OR "quality of life" OR "Self Management" OR "Self-Management" OR "Self Care" OR "Self-Care" OR "adverse outcome" OR "adverse event" OR Rehospitalization OR "patient readmission" OR readmission) | 1,014,593 |
| 4 | LA english |  |
| 5 | #1 AND #2 AND #3 AND #4 | 509 |

**web of science ([784](https://webofscience.clarivate.cn/wos/woscc/summary/be9c040f-7057-49eb-a30c-c06ad4d4de2d-0161470d70/relevance/1))**

| # | Search | Result |
| --- | --- | --- |
| 1 | TS=(postpartum period OR postnatal OR postpartum OR postpartum women OR puerperium) | [46,447](https://webofscience.clarivate.cn/wos/woscc/summary/844d116f-1f61-4b39-a695-22ef7c64284f-016146aa1e/relevance/1) |
| 2 | TS=(Discharge Planning OR Discharge Plannings OR patient discharge OR hospital discharge OR Discharge instructions OR Discharge education) | [75,153](https://webofscience.clarivate.cn/wos/woscc/summary/944695a3-2224-4d43-9cdb-ecc798e3099c-016146b7a5/relevance/1) |
| 3 | TS=("postpartum recovery" OR "postpartum complications" OR "infection" OR "mental health" OR "Mental Hygiene" OR "Depression, Postpartum" OR "postnatal depression" OR "depression" OR "anxiety" OR "breast feeding" OR "Breastfeeding" OR "infant health" OR "Baby Health" OR "Newborn Health" OR "Neonatal Health" OR "patient satisfaction" OR "satisfaction" OR "health services utilization" OR "health care utilization" OR "unmet needs" OR "Life Quality" OR "Health-Related Quality Of Life" OR "HRQOL" OR "quality of life" OR "Self Management" OR "Self-Management" OR "Self Care" OR "Self-Care" OR "adverse outcome" OR "adverse event" OR "Rehospitalization" OR "patient readmission" OR "readmission") | [1,351,391](https://webofscience.clarivate.cn/wos/woscc/summary/e49278b4-5eb1-40d2-af84-3a550931a7ef-016146e1dc/relevance/1) |
| 4 | #1 AND #2 AND #3 | 824 |
| 5 | LA=(English) |  |
| 6 | #4 AND #5 | [784](https://webofscience.clarivate.cn/wos/woscc/summary/be9c040f-7057-49eb-a30c-c06ad4d4de2d-0161470d70/relevance/1) |

**Wan Fang(1,152)**

| # | Search |  |
| --- | --- | --- |
| 1 | 产后 OR 产妇产后 OR 产褥期 OR 产褥期妇女 OR 产妇产褥期 OR 产褥期护理 OR 产后妇女 | 196,579 |
| 2 | 出院准备 OR 出院指导 OR 出院计划 OR 出院教育 | 57,369 |
| 3 | 产后恢复 OR 并发症 OR 感染 OR 心理健康 OR 产后抑郁 OR 焦虑 OR 母乳喂养 OR 婴儿健康 OR新生儿健康 OR 患者满意度 OR 满意度 OR 医疗服务利用 OR 医疗保健利用 OR 未满足的需求 OR 生活质量 OR 自我护理能力 OR 自我护理知识 OR 自我护理技能 OR 自我管理 OR 不良事件 OR 再入院 OR 患者再入院 OR 重新入院 OR 再次入院 | 4,345,497 |
| 4 | #1 AND #2 AND #3 | 1,152 |

**SinoMed([415](javascript:void(0);))**

| # | Search | Result |
| --- | --- | --- |
| 1 | 产后 OR 产妇产后 OR 产褥期 OR 产褥期妇女 OR 产妇产褥期 OR 产褥期护理 OR 产后妇女 | [138994](javascript:void(0);) |
| 2 | 出院准备 OR 出院指导 OR 出院计划 OR 出院教育 | [33516](javascript:void(0);) |
| 3 | 产后恢复 OR 并发症 OR 感染 OR 心理健康 OR 产后抑郁 OR 焦虑 OR 母乳喂养 OR 婴儿健康 OR新生儿健康 OR 患者满意度 OR 满意度 OR 医疗服务利用 OR 医疗保健利用 OR 未满足的需求 OR 生活质量 OR 自我护理能力 OR 自我护理知识 OR 自我护理技能 OR 自我管理 OR 不良事件 OR 再入院 OR 患者再入院 OR 重新入院 OR 再次入院 | [9762964](javascript:historyLink('(%20) |
| 4 | #1 AND #2 AND #3 | [415](javascript:void(0);) |

**Embase(938)**

| # | Search | Result |
| --- | --- | --- |
| 1 | 'puerperium'/exp | 95104 |
| 2 | 'post partum period':ab,ti OR 'postpartum period':ab,ti OR puerperal:ab,ti OR 'postpartum women':ab,ti OR puerperium:ab,ti | 40647 |
| 3 | #1 OR #2 | 111,582 |
| 4 | 'hospital discharge'/exp | 210,114 |
| 5 | 'patient discharge':ab,ti OR 'discharge planning':ab,ti OR 'discharge instructions':ab,ti OR 'discharge education':ab,ti OR 'hospital discharge':ab,ti | [71,332](http://www.embase.j.sjuku.top/) |
| 6 | #4 OR #5 | 229,115 |
| 7 | 'mental health'/exp | 305635 |
| 8 | 'postnatal depression'/exp | 18159 |
| 9 | 'breast feeding'/exp | 75904 |
| 10 | child health'/exp | 39496 |
| 11 | 'quality of life'/exp | 766498 |
| 12 | 'patient satisfaction'/exp | 189507 |
| 13 | 'self care'/exp | 116505 |
| 14 | 'hospital readmission'/exp | 118968 |
| 15 | 7 OR 8 OR 9 OR 10 OR 11 OR 12 OR 13 OR 14 | 1501277 |
| 16 | 'postpartum recovery':ab,ti OR 'postpartum complications':ab,ti OR infection:ab,ti | 1938280 |
| 17 | 'mental health':ab,ti OR 'mental hygiene':ab,ti OR 'postpartum depression':ab,ti OR depression:ab,ti OR anxiety:ab,ti | 1095386 |
| 18 | 'breast feeding':ab,ti OR 'infant health':ab,ti OR 'child health':ab,ti OR 'patient satisfaction':ab,ti OR satisfaction:ab,ti OR 'health services utilization':ab,ti OR 'health care utilization':ab,ti OR 'unmet needs':ab,ti OR 'health related quality of life':ab,ti OR hrql:ab,ti | 467349 |
| 19 | 'quality of life':ab,ti OR 'self management':ab,ti OR selfcare:ab,ti OR 'self care':ab,ti OR 'adverse outcome':ab,ti OR 'adverse event':ab,ti OR 'patient readmission':ab,ti OR readmission:ab,ti OR rehospitalization:ab,ti | 901619 |
| 20 | #16 OR #17 OR #18 OR #19 | 4025517 |
| 21 | #15 OR #20 | 4543240 |
| 22 | #3 AND #6 AND #20 | 986 |
| 23 | english:la |  |
| 24 | #22 AND #23 | 938 |

**Cochrane Library(775)**

| # | Search | Result |
| --- | --- | --- |
| #1 | MeSH descriptor: [Postpartum Period] explode all trees | 2787 |
| #2 | (puerperium):ti,ab,kw OR (postpartum period):ti,ab,kw OR (postpartum women):ti,ab,kw OR (postpartum):ti,ab,kw OR (postnatal):ti,ab,kw | 20795 |
| #3 | #1 OR #2 | 21270 |
| #4 | MeSH descriptor: [Patient Discharge] explode all trees | 2693 |
| #5 | (Discharge Planning):ti,ab,kw OR (Discharge Plannings):ti,ab,kw OR (patient discharge):ti,ab,kw OR (hospital discharge):ti,ab,kw OR (Discharge instructions):ti,ab,kw | 37840 |
| #6 | (Discharge education):ti,ab,kw | 3929 |
| #7 | #4 OR #5 OR #6 | 38273 |
| #8 | MeSH descriptor: [Mental Health] explode all trees | 3428 |
| #9 | MeSH descriptor: [Depression, Postpartum] explode all trees | 1081 |
| #10 | MeSH descriptor: [Breast Feeding] explode all trees | 2889 |
| #11 | MeSH descriptor: [Infant Health] explode all trees | 102 |
| #12 | MeSH descriptor: [Patient Satisfaction] explode all trees | 15538 |
| #13 | MeSH descriptor: [Quality of Life] explode all trees | 44511 |
| #14 | MeSH descriptor: [Self Care] explode all trees | 7833 |
| #15 | MeSH descriptor: [Self-Management] explode all trees | 1378 |
| #16 | MeSH descriptor: [Patient Readmission] explode all trees | 1631 |
| #17 | #8 OR #9 OR #10 OR #11 OR #12 OR #13 OR #14 OR #16 | 71432 |
| #18 | ("postpartum recovery"):ti,ab,kw OR ("postpartum complications"):ti,ab,kw OR (infection):ti,ab,kw OR ("mental health"):ti,ab,kw OR ("mental hygiene"):ti,ab,kw | 148165 |
| #19 | ("postpartum depression"):ti,ab,kw OR ("postnatal depression"):ti,ab,kw OR (depression):ti,ab,kw OR (anxiety):ti,ab,kw OR ("breast feeding"):ti,ab,kw | 158385 |
| #20 | ("breastfeeding"):ti,ab,kw OR ("infant health"):ti,ab,kw OR ("Baby Health"):ti,ab,kw OR ("Newborn Health"):ti,ab,kw OR ("Neonatal Health"):ti,ab,kw | 9604 |
| #21 | ("patient satisfaction"):ti,ab,kw OR (satisfaction):ti,ab,kw OR ("health services utilization"):ti,ab,kw OR ("health care utilization"):ti,ab,kw OR (“unmet needs”):ti,ab,kw | 84127 |
| #22 | ("Life Quality"):ti,ab,kw OR ("Health-Related Quality Of Life"):ti,ab,kw OR ("HRQOL"):ti,ab,kw OR ("quality of life"):ti,ab,kw OR ("Self Management"):ti,ab,kw | 182952 |
| #23 | ("Self-Management"):ti,ab,kw OR ("Self Care"):ti,ab,kw OR ("Self-Care"):ti,ab,kw OR ("adverse outcome"):ti,ab,kw OR ("adverse event"):ti,ab,kw | 57203 |
| #24 | (Rehospitalization):ti,ab,kw OR ("patient readmission"):ti,ab,kw OR (readmission):ti,ab,kw | 10792 |
| #25 | #18 OR #19 OR #20 OR #21 OR #22 OR #23 OR #24 | 516197 |
| #26 | #17 OR #25 | 518678 |
| #27 | #3 AND #7 AND #26 | 791 |
| #28 | English:la | 2131129 |
| #29 | #27 AND #28 | 775 |

**CNKI (75)**

| # | Search | Result |
| --- | --- | --- |
| 1 | 产后 + 产妇产后 + 产褥期 + 产褥期妇女 + 产妇产褥期 + 产褥期护理 + 产后妇女 | 11.59万 |
| 2 | 出院准备 + 出院指导 + 出院计划 + 出院教育 | 9986 |
| 3 | 产后恢复 + 并发症 + 感染 + 心理健康 + 产后抑郁 + 焦虑 + 母乳喂养 + 婴儿健康 +新生儿健康 + 患者满意度 + 满意度 + 医疗服务利用 + 医疗保健利用 + 未满足的需求 + 生活质量 + 自我护理能力 + 自我护理知识 + 自我护理技能 + 自我管理 + 不良事件 + 再入院 + 患者再入院 + 重新入院 + 再次入院 | 227.87万 |
| 4 | #1 AND #2 AND #3 | 75 |

**Psyinfo(167)**

| # | Search | Result |
| --- | --- | --- |
| 1 | MA (DE "Postnatal Period" OR DE "Perinatal Period") | 12294 |
| 2 | XB ("postpartum period" OR postnatal OR postpartum OR "postpartum women" OR puerperium OR "postnatal period" OR "perinatal period") | 37,796 |
| 3 | #1 OR #2 | 41,350 |
| 4 | MA (DE "Discharge Planning" OR DE "Hospital Discharge") | 4,017 |
| 5 | XB (discharge planning OR hospital discharge OR patient discharge OR discharge instructions OR discharge education) | 12,231 |
| 6 | #4 OR #5 | 13,607 |
| 7 | MA (DE "Mental Health" OR DE "Postpartum Depression" OR DE "Breast Feeding" OR DE "Patient Satisfaction" OR DE "Quality of Life" OR DE "Health Related Quality of Life" OR DE "Life Satisfaction" OR DE "Self-Care" OR DE "Self-Management") | 221,735 |
| 8 | XB ("postpartum recovery" OR "postpartum complications" OR "infection" OR "mental health" OR "Mental Hygiene" OR "Depression, Postpartum" OR "postnatal depression" OR "depression" OR "anxiety" OR "breast feeding" OR "Breastfeeding" OR "infant health" OR "Baby Health" OR "Newborn Health" OR "Neonatal Health" OR "patient satisfaction" OR "satisfaction" OR "health services utilization" OR "health care utilization" OR "unmet needs" OR "Life Quality" OR "Health-Related Quality Of Life" OR "HRQOL" OR "quality of life" OR "Self Management" OR "Self-Management" OR "Self Care" OR "Self-Care" OR "adverse outcome" OR "adverse event" OR "Rehospitalization" OR "patient readmission" OR "readmission") | 878,090 |
| 9 | #7 OR #8 | 912,344 |
| 10 | LA english |  |
| 11 | #3 AND #6 AND #9 AND #10 | 167 |
