# Supplementary material for: Effectiveness of discharge planning interventions on health-related outcomes among postpartum women: a systematic review and meta-analysis
Source: Front Public Health. 2026 Mar 23;14:1733799. doi: 10.3389/fpubh.2026.1733799 (PMC13051705; doi:10.3389/fpubh.2026.1733799)
Supplement: Supplementary file 2 [file Table_2.docx]

| **Study ID** | **Reference Number** | **Country & Type** | **Participants, n** | **Intervention** | **Intervention strategie** | **Evaluation time points** | **Main outcomes** |
| --- | --- | --- | --- | --- | --- | --- | --- |
| Wang 2007 | [23] | China /  Quasi-experimental | 750  I: 382  C: 368 | Intensive breastfeeding education | E | Prior to discharge、  4 months postpartum | Breastfeeding Knowledge、  Breastfeeding Rate、  Health Education Satisfaction Rate |
| Xia 2013 | [30] | China / RCT | 60  I: 30  C: 30 | Professional Skills Guidance Table | E | Prior to discharge | Breastfeeding Skills、Newborn care skills、Healthcare Skills Mastery Rate、Nursing Satisfaction Rate |
| Xie 2010 | [24] | China / RCT | 300  I: 150  C: 150 | Clinical Nursing Pathway Health Education | E | Prior to discharge | Breastfeeding Theory Examination、Breastfeeding Success Rate、Nursing Service Satisfaction |
| Yang 2010 | [31] | China / RCT | 740 primipara  I: 390  C: 350 | Targeted health education | E | Prior to discharge | Self-Care Knowledge、  Exclusive Breastfeeding、  Nursing Service Satisfaction |
| Yao(1) 2021 | [32] | China / RCT | 128 primipara  I: 64  C: 64 | Feeding education based on the behavior change model | E + T | 42 days postpartum | Breastfeeding Self-Efficacy、  Exclusive Breastfeeding Rate、  Health Education Satisfaction Rate |
| Yao(2) 2021 | [25] | China / RCT | 100 primipara  I: 50  C: 50 | Health education based on the behavior change model | E + T + P | 6 weeks postpartum | Nutrient Intake、  Lactation Status、  Breastfeeding Rate |
| Yu 2024 | [33] | China / RCT | 102  I: 51  C: 51 | Personalized health guidance based on psychological adjustment | E + T | After two weeks of intervention | Anxiety、Depression、Rehabilitation Treatment Compliance |
| Zhou 2016 | [34] | China / RCT | 120  I: 60  C: 60 | Stage-based health education | E + T | Prior to discharge | Self-care ability、Postoperative Complications、Breastfeeding Rate、Anxiety、Depression |
| Zhu 2017 | [35] | China / RCT | 200  I: 100  C:100 | Collaborative Care | E + T + P | Prior to discharge | Anxiety、  Maternal-Infant Care Knowledge、Nursing Satisfaction |
| Ahmed 2016 | [37] | USA / RCT | 106  I: 49  C: 57 | Web-based interactive monitoring | E + T + P | At discharge  1、2 and 3 months after discharge | Exclusive breastfeeding rate、  any breastfeeding rate、  postpartum depression |
| Bick 2012 | [43] | UK /  Quasi-experimental | 1466  I: 725  C: 741 | Continuous quality improvement method | E + T | 10 days postpartum、  3 months postpartum | breastfeeding、Maternal morbidity、Depression、Nursing Satisfaction |
| Çankaya 2024 | [39] | Türkiye / RCT | 128 primipara  I: 64  C: 64 | Carry out labor care in accordance with the recommendations of the World Health Organization (WHO) | E + T | postpartum | Maternal parenting behaviors、Breastfeeding self-efficacy、  breastfeeding assessment tool |
| Consales 2022 | [26] | Italy /  Quasi-experimental | 460  I: 269  C: 191 | Carry out postpartum education for mothers based at the hospital | E + T | At discharge  48 hours post-discharge | Exclusive breastfeeding rate、  Nurse-Parent Support Tool |
| Dodt 2015 | [42] | Brazil /  Quasi-experimental | 96  I: 54  C: 42 | Carry out postpartum education for mothers based at the hospital | E + T | 2 months postpartum | Breastfeeding self-efficacy、  Duration of breastfeeding |
| Estalella 2020 | [27] | Spain /  Quasi-experimental | 373 preterm parturient  I: 161  C: 212 | Postpartum Handbook | E + T | At discharge | Breastfeeding rate、  Breast pump utilization rate、  Morbidity rate、  Length of hospital stay |
| Fu  2014 | [28] | China / RCT | 722 primipara  I_1_: 191  I_2_: 268  C: 263 | I_1_:Hospitalization support  I_2_: Postpartum telephone follow-up support | E + T | 1, 2, and 3 months postpartum | Any breastfeeding、  Exclusive breastfeeding |
| Gao 2015 | [36] | China / RCT | 180 primipara  I: 90  C: 90 | Postpartum education oriented towards interpersonal psychotherapy | E + T | 6 weeks postpartum | Breastfeeding status、Postpartum depression、Perceived social  support、Maternal role competence |
| Gozali 2020 | [38] | USA /  Quasi-experimental | 84 primipara  I: 36  C: 48 | Pediatrician-led newborn parenting course | E | At discharge | Anxiety、  Parenting confidence、  Neonatal care knowledge |
| Kashyap 2022 | [41] | India /  Quasi-experimental | 4984  I: 3510  C: 1474 | Nursing Companion Program | E + T | 2 weeks postpartum | Exclusive breastfeeding、Infant care skills、Neonatal complications、Maternal complications、Neonatal readmission rate |
| Johnston 2025 | [29] | India / RCT | 21937  I: 11611  C: 10326 | Receive mobile information services through the WhatsApp platform | E + T + P | 8 weeks postpartum | Exclusive breastfeeding、 Maternal dietary behaviors、Neonatal complications、Maternal complications、Readmission rate |
| Yilmaz 2020 | [40] | Türkiye /  Quasi-experimental | 60  I: 30  C: 30 | Kangaroo mother care | E | 3 days postpartum | Breastfeeding self-efficacy、  Perceived insufficient milk supply |

**Supplementary File S2.** Basic information of eligible literature.

Note: RCT: randomized control trial; I: intervention group; C: control group. E = Predischarge Education;

T = Post-discharge Telephone Follow-up; P = Information Exchange Platform.

**The references included in the study:**

23. Wang X, Zhou Y, and Zhou Y. Effect of Enhanced Perioperative Health Education on Breastfeeding in Cesarean Section. Journal of Medical Information. (2007) 05: 831-832.

24. Xie A, Fan J, and Tang Y. Application of clinical nursing path in the health education of elective caesarean section. CHINA MEDICAL HERALD. (2010) 7: 74-75.

25. Yao L, Yu Y, He Y, and Sheng J. The influence of health education based on the behavioral change model on postpartum nutritional intake, milk secretion and breastfeeding behavior of mothers. Maternal and Child Health Care of China. (2021) 36: 4835-4838. doi: 10.19829/j.zgfybj.issn.1001-4411.2021.20.062

26. Consales A, Colombo L, Zanotta L, Morniroli D, Sannino P, Rampini S, et al. Pilot Feasibility Study of a Hospital-Based Post-Natal Educational Intervention on New Mothers in a BFHI-Compliant Tertiary Referral Center for Neonatal Care. International journal of environmental research and public health. (2022) 19 doi: 10.3390/ijerph19042020

27. Estalella I, San Millán J, Trincado MJ, Maquibar A, Martínez-Indart L, and San Sebastián M. Evaluation of an intervention supporting breastfeeding among late-preterm infants during in-hospital stay. Women and birth : journal of the Australian College of Midwives. (2020) 33: e33-e38. doi: 10.1016/j.wombi.2018.11.003

28. Fu IC, Fong DY, Heys M, Lee IL, Sham A, and Tarrant M. Professional breastfeeding support for first-time mothers: a multicentre cluster randomised controlled trial. BJOG. (2014) 121: 1673-83. doi: 10.1111/1471-0528.12884

29. Johnston JS, Suri P, Yan S, Chandrasekar A, Singla S, Ward VC, et al. A mobile messaging service for families on postnatal knowledge and practices: a cluster randomized trial, India. Bulletin of the World Health Organization. (2025) 103: 255-265. doi: 10.2471/blt.24.292145

30. Xia Q, and Sun J. Application of professional skills guidance table in health education after cesarean section. Chinese Nursing Research. (2013) 27: 921-922.

31. Yang J, and Li Y. The Application of Goal-Based Health Education in Pregnant Women. Hainanme Dical Journal. (2010) 21: 153-155.

32. Yao L, Han P, and He Y. Effect of Feeding Education Based on the Behavior Change Model on Breastfeeding Confidence and Feeding Behaviors in Primiparous Women. Maternal and Child Health Care of China. (2021) 36: 5806-5809. doi: 10.19829/j.zgfybj.issn.1001-4411.2021.24.060

33. Yu Y, Du Y, and Fang F. The influence of a personalized health guidance program based on psychological regulation on the treatment compliance of postpartum psychological state and pelvic floor function of parturients. Maternal and Child Health Care of China. (2024) 39: 535-538. doi: 10.19829/j.zgfybj.issn.1001-4411.2024.03.037

34. Zhou Y. Evaluation on the Clinical Effect of Staged Health Education During Perinatal Period of Cesarean Section. China Continuing Medical Education. (2016) 8: 181-183.

35. Zhu H. The Application of Collaborative Care in Mother-Baby Rooming-Together Setup. Women's Health Research. (2017): 91+128.

36. Gao LL, Xie W, Yang X, and Chan SW. Effects of an interpersonal-psychotherapy-oriented postnatal programme for Chinese first-time mothers: a randomized controlled trial. International journal of nursing studies. (2015) 52: 22-9. doi: 10.1016/j.ijnurstu.2014.06.006

37. Ahmed AH, Roumani AM, Szucs K, Zhang L, and King D. The Effect of Interactive Web-Based Monitoring on Breastfeeding Exclusivity, Intensity, and Duration in Healthy, Term Infants After Hospital Discharge. Journal of obstetric, gynecologic, and neonatal nursing : JOGNN. (2016) 45: 143-54. doi: 10.1016/j.jogn.2015.12.001

38. Gozali A, Gibson S, Lipton LR, Pressman AW, Hammond BS, and Dumitriu D. Assessing the effectiveness of a pediatrician-led newborn parenting class on maternal newborn-care knowledge, confidence and anxiety: A quasi-randomized controlled trial. Early human development. (2020) 147: 105082. doi: 10.1016/j.earlhumdev.2020.105082

39. Çankaya S, Tezgören E, and Dikmen HA. The effects of the intrapartum care model given in line with the recommendations of the World Health Organization (WHO) on the mother's maternal behavior towards her baby, breastfeeding self-efficacy, breastfeeding success, and hospital discharge readiness: a randomized controlled trial. Arch. Gynecol. Obstet. (2024) 310: 3009-3027. doi: 10.1007/s00404-024-07844-0

40. Yilmaz F, Küçükoğlu S, Aytekin Özdemir A, Oğul T, and Aşki N. The Effect of Kangaroo Mother Care, Provided in the Early Postpartum Period, on the Breastfeeding Self-Efficacy Level of Mothers and the Perceived Insufficient Milk Supply. The Journal of perinatal & neonatal nursing. (2020) 34: 80-87. doi: 10.1097/jpn.0000000000000434

41. Kashyap S, Spielman AF, Ramnarayan N, Sd S, Pant R, Kaur B, et al. Impact of family-centred postnatal training on maternal and neonatal health and care practices in district hospitals in two states in India: a pre-post study. BMJ open quality. (2022) 11 doi: 10.1136/bmjoq-2021-001462

42. Dodt RC, Joventino ES, Aquino PS, Almeida PC, and Ximenes LB. An experimental study of an educational intervention to promote maternal self-efficacy in breastfeeding. Revista latino-americana de enfermagem. (2015) 23: 725-32. doi: 10.1590/0104-1169.0295.2609

43. Bick D, Murrells T, Weavers A, Rose V, Wray J, and Beake S. Revising acute care systems and processes to improve breastfeeding and maternal postnatal health: a pre and post intervention study in one English maternity unit. BMC pregnancy and childbirth. (2012) 12: 41. doi: 10.1186/1471-2393-12-41
